# On the Validity and Phylogenetic Position of *Eubrachiosaurus browni,* a Kannemeyeriiform Dicynodont (Anomodontia) from Triassic North America

**DOI:** 10.1371/journal.pone.0064203

**Published:** 2013-05-31

**Authors:** Christian F. Kammerer, Jörg Fröbisch, Kenneth D. Angielczyk

**Affiliations:** 1 Museum für Naturkunde, Leibniz Institute for Research on Evolution and Biodiversity, Berlin, Germany; 2 Department of Geology, Field Museum of Natural History, Chicago, Illinois, United States of America; Raymond M. Alf Museum of Paleontology, United States of America

## Abstract

The large dicynodont *Eubrachiosaurus browni* from the Upper Triassic Popo Agie Formation of Wyoming is redescribed. *Eubrachiosaurus* is a valid taxon that differs from *Placerias hesternus*, with which it was previously synonymized, by greater anteroposterior expansion of the scapula dorsally and a very large, nearly rectangular humeral ectepicondyle with a broad supinator process. Inclusion of *Eubrachiosaurus* in a revised phylogenetic analysis of anomodont therapsids indicates that it is a stahleckeriid closely related to the South American genera *Ischigualastia* and *Jachaleria*. The recognition of *Eubrachiosaurus* as a distinct lineage of North American dicynodonts, combined with other recent discoveries in the eastern USA and Europe, alters our perception of Late Triassic dicynodont diversity in the northern hemisphere. Rather than being isolated relicts in previously therapsid-dominated regions, Late Triassic stahleckeriid dicynodonts were continuing to disperse and diversify, even in areas like western North America that were otherwise uninhabited by coeval therapsids (i.e., cynodonts).

## Introduction

The Triassic is generally considered a time of diminishing fortunes for the synapsid lineage [1], [2]. Compared to their Permian heyday, Triassic synapsids were relatively species-poor and ecologically restricted. The canonical example of this trend is the anomodont subclade Dicynodontia. Once the dominant Permian terrestrial herbivores in terms of abundance, species richness, and ecological diversity, only four lineages of dicynodonts survived the Permo-Triassic extinction [3], [4]. Of these four, two (Lystrosauridae and Myosauridae) were post-extinction “disaster taxa” that did not survive beyond the Early Triassic [5] and one (Kingoriidae) was extremely rare (*Kombuisia*, the sole Triassic genus, is known from four specimens [6], [7]). Only the Kannemeyeriiformes underwent a significant diversification during the Triassic, with roughly 40 known species [4], [8]. Unlike Permian anomodonts, however, which are known from ∼90 species spanning mouse-to-rhinoceros sizes and occupying an array of niches (including fossorial and arboreal forms) [9], [10], all kannemeyeriiforms were medium- to large-bodied [11], graviportal herbivores with relatively erect posture and gait [12], [13].

**Figure 1:**
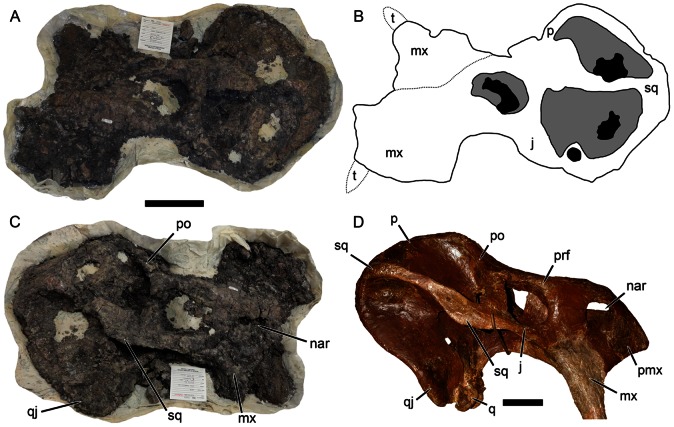
Reinterpretation of an articulated skull of *Placerias hesternus* (MNA V8464). (A) specimen as oriented by Lucas and Heckert [18] (anterior left, dorsal up); (B) interpretive drawing of A based on the previous interpretation [18]; (C) our reinterpretation of the specimen (anterior right, dorsal up), comparable to (D) the composite reconstruction of *Placerias* (UCMP 137369) by Camp and Welles [23] based on disarticulated material from the *Placerias* Quarry. Note similar flange on the squamosal contribution to the zygomatic arch in C and D. The irregular mass considered the left maxilla in B represents indeterminate bone fragments in matrix. *Abbreviations*: *j*, jugal; *mx*, maxilla; *nar*, external naris; *po*, postorbital; *prf*, prefrontal; *q*, quadrate; *qj*, quadratojugal; *sq*, squamosal; *t*, tusk. Scale bars equal 10 cm. [formatted for 2 column width].

Unlike cynodonts, which show a primarily Gondwanan distribution [14], kannemeyeriiform dicynodonts were distributed worldwide in the Middle Triassic [4]. In addition to the well-known dicynodont records of South America and Africa, assemblages with multiple sympatric kannemeyeriiform taxa are known from the Anisian of Russia and China [15], [16]. Fitting the concept of progressive synapsid decline, Late Triassic kannemeyeriiforms are by contrast present in low abundance, and in most assemblages where they occur only a single species is present. They are also thought to be geographically restricted: until recently only a single Late Triassic dicynodont species was known outside of South America. That species, *Placerias hesternus*, is usually listed as the only Late Triassic North American dicynodont [17], [18]. *Placerias* is best known from the *Placerias* Quarry in eastern Arizona (Blue Mesa Member, Chinle Formation), which yielded roughly 1600 *Placerias* elements representing at least 41 individuals [19], [20]. However, this is an exceptional case and may represent a drought-driven concentration of individuals [19]: outside the *Placerias* Quarry this taxon is extremely rare, with only a handful of isolated elements known from less than 10 other localities in the American southwest [18] and North Carolina [21], [22]. Of these, the most significant specimen is a relatively complete skull (MNA V8464) that was briefly described by Lucas and Heckert [18], although they oriented the specimen upside down (Figures 1A, 1B). When the specimen is properly oriented (Figure 1C), it bears a striking resemblance to the hypothetical reconstruction of the skull developed by Camp and Welles [23] and modified by Cox [24] (Figure 1D). As such, this specimen confirms the accuracy of the Camp and Welles/Cox reconstruction, which has been used extensively in studies of the systematic position of *Placerias* [25]–[27].

However, *Placerias* is not the only dicynodont described from the Late Triassic of North America. Williston [28] described two dicynodont genera from the Popo Agie Formation of Wyoming, *Eubrachiosaurus browni* and *Brachybrachium brevipes*. Lucas and Hunt [17] considered these taxa junior synonyms of *Placerias hesternus*, a position maintained in most subsequent studies (e.g., [18]). However, Long and Murry [29] questioned the synonymy of *Eubrachiosaurus* and *Placerias*, noting that the ectepicondyle of *Eubrachiosaurus* was enlarged as in *Ischigualastia*.

Unfortunately, most of Williston’s dicynodont material from Popo Agie has been lost, hindering restudy of this taxon. Of the holotype of *Eubrachiosaurus browni* (FMNH UC 633), the humerus and pelvis have been lost, only the scapula remains. The holotypic partial humerus of *Brachybrachium brevipes* has been lost entirely. Searches of the Field Museum and University of Chicago (these specimens were originally housed at the University of Chicago Walker Museum) have failed to locate any of these missing specimens and it is probable that they were discarded or destroyed prior to the transfer between institutions. Fortunately, and unusually for the time period, Williston [28] published photographs of these elements, so we can be confident of their morphology. In this contribution we redescribe *Eubrachiosaurus browni* based on study of the remaining portion of the holotype and the original photographs, address its supposed synonymy with *Placerias*, and investigate the phylogenetic position of the taxon and its implications for Late Triassic dicynodont diversity.

## Materials and Methods

We compared the surviving and photographed material of problematic North American kannemeyeriiforms (*Eubrachiosaurus browni*, *Brachybrachium brevipes*, NMMNH P-13001) with kannemeyeriiform postcranial material we have examined firsthand: *Angonisaurus cruickshanki* (NHMUK R9732); *Dinodontosaurus pedroanum* (MCN 3584, MCP 130, MCP 4172, MCZ 1670, 1687, 3108, 3454, UFRGS PV0115T, PV0116T, PV0161T); *Dolichuranus primaevus* (CGP/1/191A); *Ischigualastia jenseni* (MACN 18055, MCZ 3119, PVL 3807, PVSJ 607); *Jachaleria candelariensis* (UFRGS PV0150T, PV0151T, PV0287T); *Kannemeyeria simocephalus* (BP/1/4523, 4550, 5624, CAMZM T757, ELM 1, NHMUK R3740, R3741, R3758, R3760, R3761, R3762, SAM-PK-2771, 3017, UCMP 38373); *Parakannemeyeria youngi* (PIN 2422/1); *Placerias hesternus* (MNA V2713, UCMP 24782, 25069, 25093, 25361, 25373, 32393, 32394, 32459, USNM 2198); *Rhadiodromus klimovi* (PIN 159/1); *Shansiodon wangi* (IVPP V2415); *Sinokannemeyeria yingchiaoensis* (IVPP V974); *Stahleckeria potens* (GPIT/RE/8001); *Tetragonias njalilus* (CAMZM T754, GPIT/RE/7110); *Wadiasaurus indicus* (ISI R175/1); *Xiyukannemeyeria brevirostris* (IVPP V4457, 4458); and *Zambiasaurus submersus* (NHMUK R9068, 9069, 9089, 9091, 9103, 9106, 9109, 9113, 9118, 9122, 9140). We also made comparisons based on descriptions from the literature [30]–[36]. No permits were required for the described study, which complied with all relevant regulations.

Insitutional Abbreviations: AMNH, American Museum of Natural History, New York, NY, USA; BP, Bernard Price Institute, University of the Witwatersrand, Johannesburg, South Africa; CAMZM, University Museum of Zoology, Cambridge, UK; CGP, Council for Geosciences, Pretoria, South Africa; ELM, East London Museum, East London, South Africa; FMNH, Field Museum of Natural History, Chicago, IL, USA; ISI, Indian Statistical Institute, Kolkata, India; IVPP, Institute for Vertebrate Paleontology and Paleoanthropology, Chinese Academy of Sciences, Beijing, China; GPIT, Institut für Geowissenschaften, Eberhard Karls Universität Tübingen, Tübingen, Germany; MACN, Museo Argentino de Ciencias Naturales “Bernardino Rivadavia”, Buenos Aires, Argentina; MCN, Museu de Ciências Naturais, Fundação Zoobotânica do Rio Grande do Sul, Porto Alegre, Brazil; MCP, Museu de Ciências e Tecnologia, Pontificia Universidade Católica do Rio Grande do Sul, Porto Alegre, Brazil; MCZ, Museum of Comparative Zoology, Harvard University, Cambridge, MA, USA; MNA, Museum of Northern Arizona, Flagstaff, AZ, USA; NCSM, North Carolina Museum of Natural Sciences, Raleigh, NC, USA; NHCC, National Heritage Conservation Commission, Lusaka, Zambia; NHMUK, The Natural History Museum, London, UK; NMMNH, New Mexico Museum of Natural History & Science, Albuquerque, NM, USA; PIN, Paleontological Institute of the Russian Academy of Sciences, Moscow, Russia; PVL, Museo Miguel Lillo de Ciencias Naturales, San Miguel de Tucumán, Argentina; PVSJ, Museo de Ciencias Naturales, Universidad Nacional de San Juan, San Juan, Argentina; SAM, Iziko, the South African Museum, Cape Town, South Africa; SGU, Saratov State University, Moscow, Russia; TSK, T. S. Kemp Collection, Oxford University, Oxford, UK; UCMP, University of California Museum of Paleontology, Berkeley, CA, USA; UFRGS, Universidade Federal do Rio Grande do Sul, Porto Alegre, Brazil; US, University of Stellenbosch, Stellenbosch, South Africa; USNM, National Museum of Natural History, Washington, DC, USA.

## Results

### Systematic Paleontology

Therapsida Broom, 1905 [37].

Anomodontia Owen, 1860 [38].

Dicynodontia Owen, 1860 [38].

Kannemeyeriiformes Maisch, 2001 [26].

Stahleckeriidae (Lehman, 1961) [39].

*Eubrachiosaurus* Williston, 1904 [28].

Syn. ?*Brachybrachium* Williston, 1904 [28].

#### ZooBank Life Science Identifier (LSID)

urn:lsid:zoobank.org:act:42BD6746-42DB-4FAA-851E-6297F43BB3EE.

#### Type species

*Eubrachiosaurus browni* Williston, 1904 [28].

#### Diagnosis

As for the type and only species.

*Eubrachiosaurus browni* Williston, 1904 [28].

Syn. ?*Brachybrachium brevipes* Williston, 1904 [28].

#### ZooBank Life Science Identifier (LSID)

urn:lsid:zoobank.org:act:0BC656A0-02E9-4CDD-884C-27EC8BEBF0B7.

#### Holotype

FMNH UC 633, originally a partial left scapula, left humerus, and left pelvis, of which only the scapula remains.

#### Type locality and Horizon

Little Popo Agie River, near Lander, Fremont County, Wyoming. Popo Agie Formation, Chugwater Group, Late Triassic (Norian).

#### Diagnosis

Kannemeyeriiform dicynodont characterized by a unique combination of postcranial characters: well-developed scapular spine; scapula strongly constricted at level of acromion; anterior and distal edges of deltopectoral crest close to perpendicular; humeral ectepicondyle large, nearly rectangular; anterior iliac blade long, curving anteroventrally. An autapomorphy of *Eubrachiosaurus* is the extreme curvature of the anteroventral margin of the iliac blade.

### Scapula

**Figure 2:**
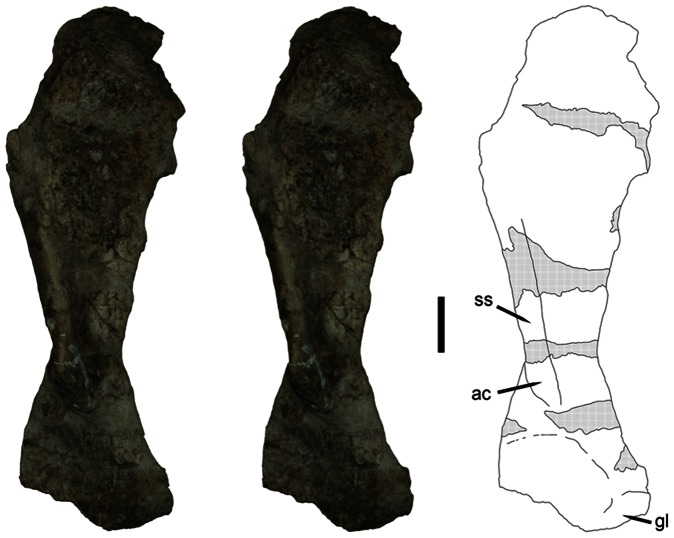
FMNH UC 633. Stereopair and interpretive drawing of the left scapula from the holotype of *Eubrachiosaurus browni* in lateral view. Hatched regions on drawing indicate areas where the specimen is reconstructed with plaster. *Abbreviations*: *ac*, acromion process; *gl*, edge of glenoid fossa; *ss*, scapular spine. Scale bar equals 5 cm. [stereogram; formatted for 2 column width].

The sole remaining element of the holotype is a left scapula (Figure 2). This specimen is badly weathered and fragmentary, with the anterior and posterior edges of the proximal portion of the scapula broken off. Several sections of the specimen down its length are reconstructed with plaster. This specimen is 48.4 cm total length along the long axis. The scapular spine is mostly reconstructed with plaster, but from the preserved sections it was clearly robust and prominent. The acromion process is worn, with only the base remaining, but the base is well developed and protrudes laterally and slightly anteriorly. The scapula is anteroposteriorly narrow (constricted) at the level of the acromion and gradually widens dorsally, although the maximum width of the dorsal portion is unknown as this region is badly damaged. Part of the lip around the glenoid fossa is preserved at the posteroventral edge of the scapula. The anteroventral portion of scapula is broadly flattened and slightly concave, as in other kannemeyeriiforms. The medial surface of the scapula is almost entirely obscured by plaster and a metal rod made to support the element.

**Figure 3:**
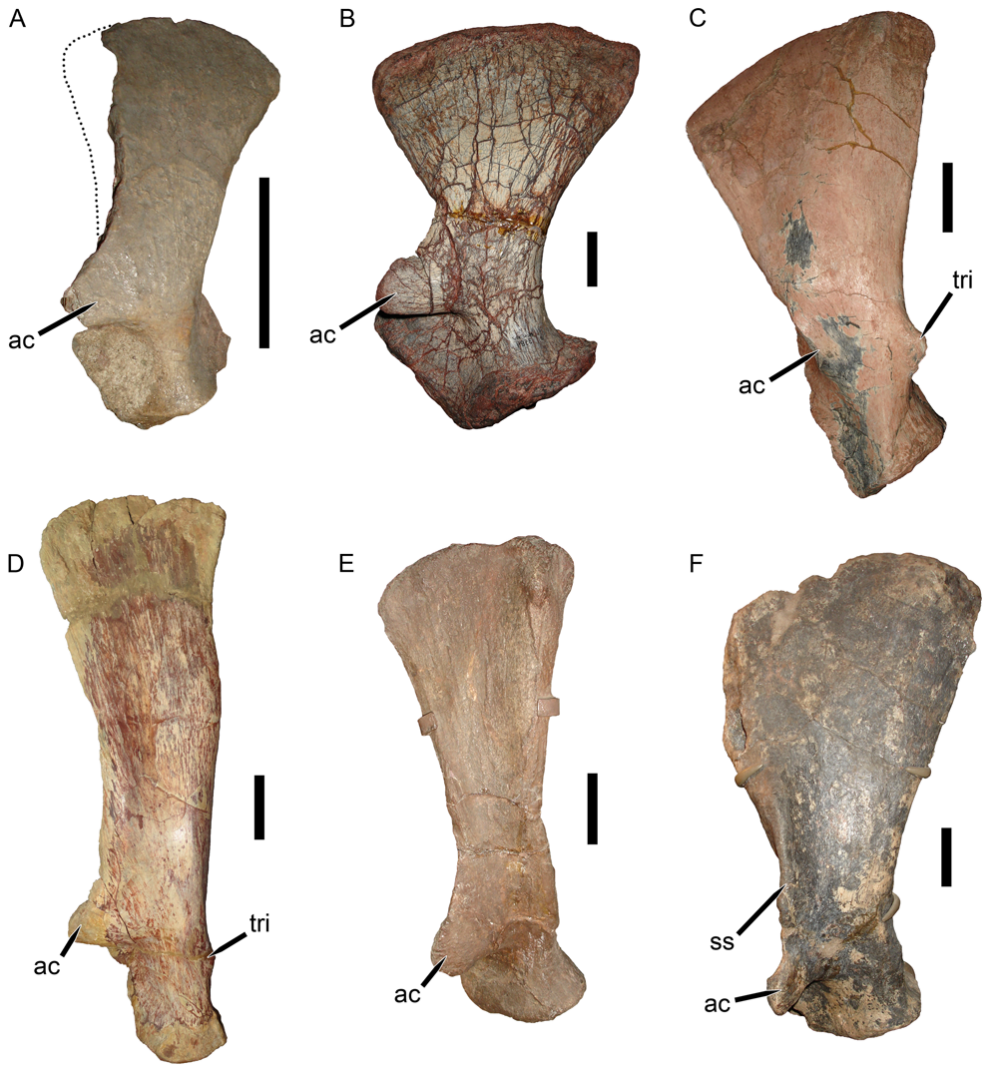
Kannemeyeriiform scapulae in lateral view. (A) IVPP V2415, *Shansiodon wangi* (dotted line indicates anterior edge of scapular blade, broken off in this specimen but restored based on the condition in *Tetragonias* and *Rhinodicynodon*); (B) MCN PV 3584, *Dinodontosaurus pedroanum*; (C) UFRGS PV-0151T, *Jachaleria candelariensis* (acromion process broken off in this specimen, arrow indicates base of process); (D) NHMUK R3740, *Kannemeyeria simocephalus*; (E) IVPP V974, *Sinokannemeyeria yingchiaoensis*; (F) GPIT/RE/8001, *Stahleckeria potens*. The specimens in A and E are left scapulae, the specimens in B, C, D, and F are right scapulae that have been reversed for comparative purposes. *Abbreviations*: *ac*, acromion process; *ss*, scapular spine; *tri*, tricipital tubercle. Scale bars equal 5 cm. [formatted for 2 column width].

**Figure 4:**
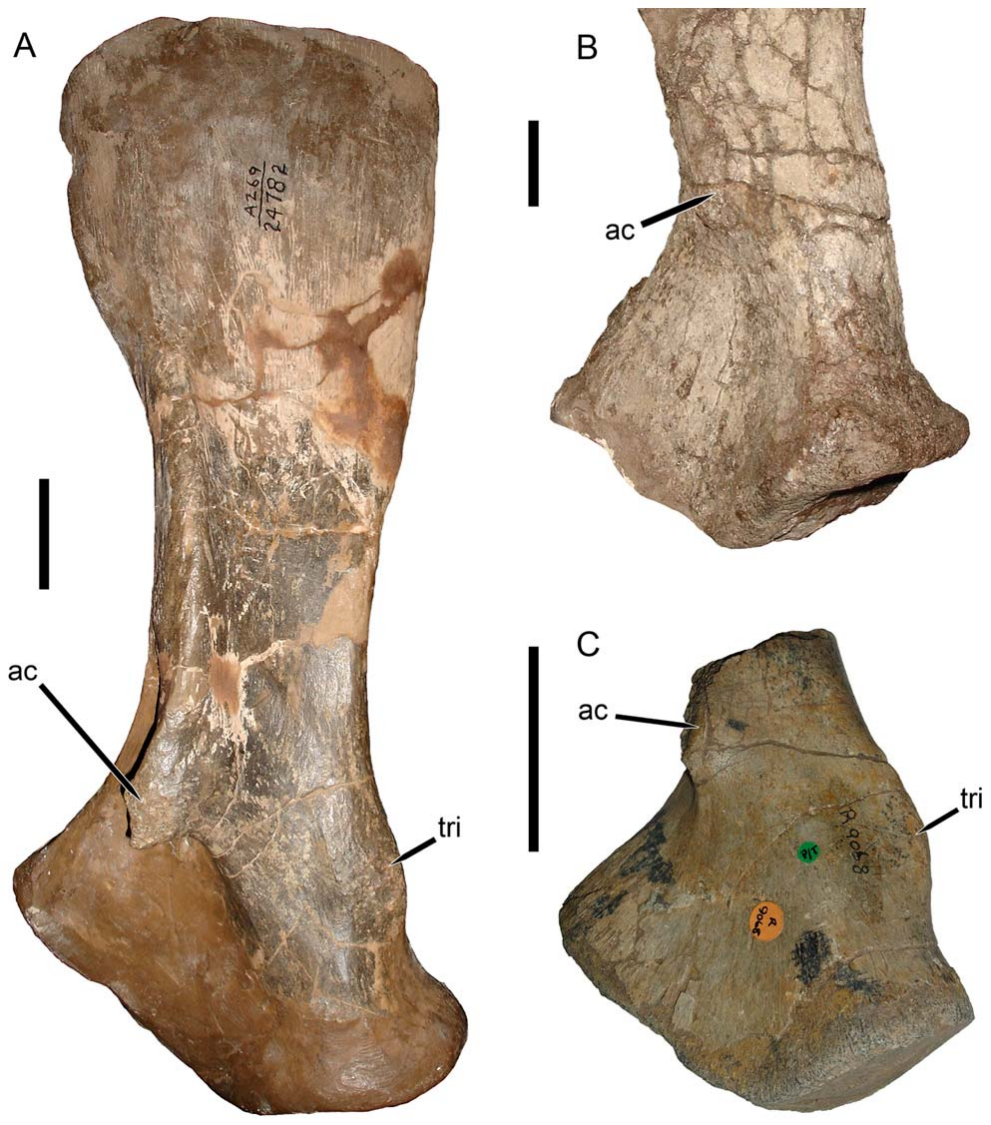
Stahleckeriid scapulae in lateral view. (A) UCMP 24782, left scapula of *Placerias hesternus* (partially reconstructed, plaster is light brown); (B) PVL 3807, distal portion of left scapula of *Ischigualastia jenseni*; (C) NHMUK R9068, distal portion of left scapula of *Zambiasaurus submersus* (acromion process broken off in this specimen, arrow indicates base of process). *Abbreviations*: *ac*, acromion process; *tri*, tricipital tubercle. Scale bars equal 5 cm. [formatted for 1.5 column width].

The poor preservation of the scapula of *Eubrachiosaurus* complicates comparisons with other taxa, but several distinctions can be made. Kannemeyeriiform scapulae can be divided into three general morphotypes (Figure 3): shansiodontids, which have a relatively large, robust, anteriorly-directed acromion process, no distinct scapular spine, and a short dorsal portion of the scapula (here referring to the entire scapular blade above the level of the acromion) which flares broadly dorsally (Figures 3A, 3B); “kannemeyeriids”, which have a relatively small, anteriorly-directed acromion process, weak, elongate scapular spine (if present), and a tall dorsal portion of the scapula with very gradual expansion dorsally (unlike the flared condition in shansiodontids) (Figures 3D, 3E); and stahleckeriids, which have a relatively small, anterolaterally-directed acromion process, tall dorsal portion of the scapula that expands broadly dorsally, and a distinct postero-anterior slope to the dorsal margin of the scapula (Figures 3C, 3F). The scapular spine is variable in stahleckeriids; in *Stahleckeria* it is well developed (Figure 3F), whereas in *Jachaleria* (Figure 3C, [33]) and *Ischigualastia* (Figure 4B, [24]) it is absent.

Although *Placerias* has been recovered as a stahleckeriid in our previous phylogenies [27], its scapular morphology is distinct from other members of the clade (Figure 4A). A strong scapular spine is present, but the morphology of the dorsal portion of the scapula is more similar to the “kannemeyeriid” condition: tall and only slightly expanded dorsally (although not to the degree of *Kannemeyeria* (Figure 3D) or *Parakannemeyeria* [30]). The scapula of *Eubrachiosaurus* has a similarly tall dorsal portion as in *Placerias*, but with more extreme dorsal expansion (especially considering that the anterior and posterior edges are broken off dorsally). The scapula is markedly more anteroposteriorly constricted at the level of the acromion process in *Eubrachiosaurus* than in *Placerias*. *Eubrachiosaurus* is similar to *Placerias* in having a long, robust scapular spine, but this morphology is also present in *Stahleckeria*. In general, the scapula of *Eubrachiosaurus* is most similar to that of *Stahleckeria* among kannemeyeriiforms, although the dorsal portion is less anteroposteriorly broad than *Stahleckeria* (even accounting for the missing edges dorsally, compare degree of dorsal expansion in Figure 2 with Figure 3F).

The scapula of *Placerias* bears a well-developed, mound-like tubercle for attachment of the triceps (Figure 4A); this type of tubercle also occurs in *Zambiasaurus* (Figure 4C). A distinct tricipital tubercle is absent in most kannemeyeriiforms. A weak tubercle occurs in *Kannemeyeria* (Figure 3D) and a sharply pointed tubercle quite unlike the broad mound of *Placerias* occurs in *Jachaleria* (Figure 3C). The relevant region is mostly reconstructed with plaster in *Eubrachiosaurus*, but there is no sign of such a tubercle at the edges, suggesting that if this feature was present it was not the mound-like structure as in *Placerias*.

### Humerus

**Figure 5:**
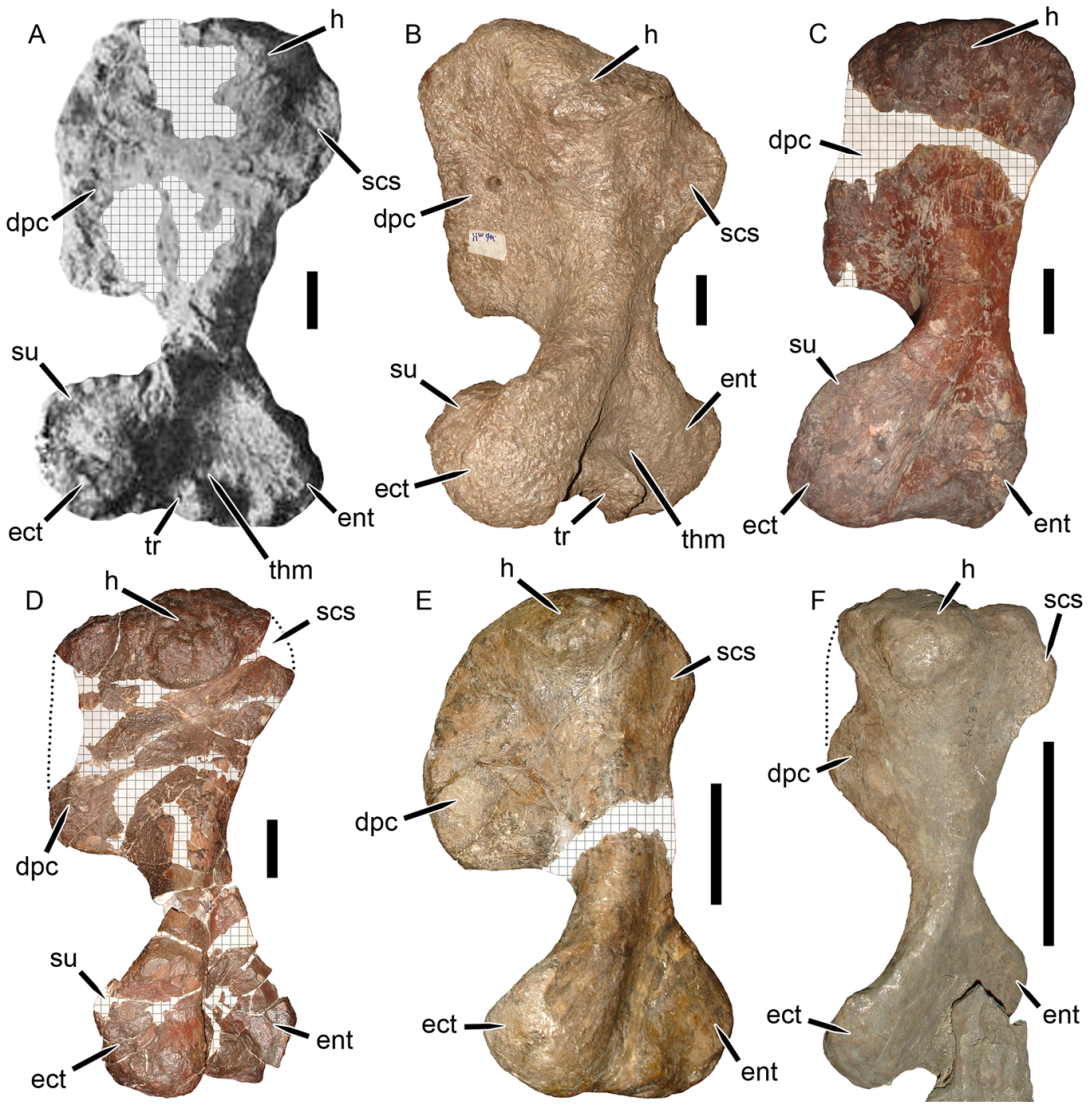
Kannemeyeriiform humeri in dorsal view. (A) lost element from the holotype of *Eubrachiosaurus browni* (modified from [28]); (B) PVL 3807, *Ischigualastia jenseni*; (C) NHMUK R3741, *Kannemeyeria simocephalus*; (D) USNM 2198, *Placerias hesternus*; (E) IVPP V4457, *Xiyukannemeyeria brevirostris*; (F) IVPP V2415, *Shansiodon wangi*. The specimens in A, C, and F are left humeri, the specimens in B, D, and E are right humeri that have been reversed for comparative purposes. Hatched areas indicate plaster reconstruction, dotted lines indicate missing portions of specimen. *Abbreviations*: *dpc*, deltopectoral crest; *ect*, ectepicondyle; *ent*, entepicondyle; *h*, humeral head; *scs*, attachment site for M. subcoracoscapularis; *su*, supinator process; *thm*, attachment site for M. triceps humeralis medialis; *tr*, trochlea. Scale bars equal 5 cm. [formatted for 2 column width].

The recovered forelimb material of *Eubrachiosaurus browni* was a largely complete left humerus, although Williston’s ([28]:Fig. 3) figure of this specimen shows that part of the proximal portion was reconstructed with plaster (see Figure 5A). Williston [28] only figured the dorsal view, and although he provided information on the ventral side, its degree of reconstruction is uncertain. The shaft of the humerus was narrow and the deltopectoral crest was massive and flared, with a distinct perpendicular angle between its proximal and distal margins. According to Williston [28], the humerus was 44.0 cm long, with 23.7 cm greatest width of the proximal portion, 6.8 cm least width of the shaft, and 26.3 cm greatest width of the distal portion.

**Figure 6:**
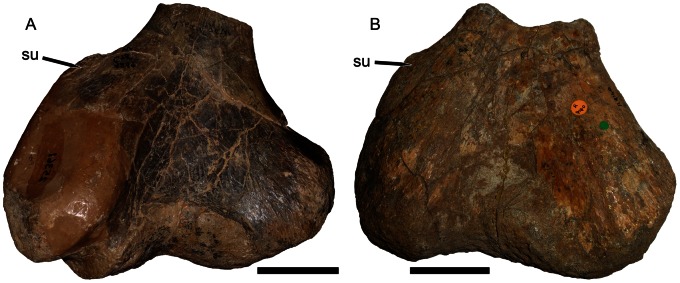
Placeriine distal humeri in dorsal view. (A) UCMP 25361, *Placerias hesternus* (right humerus reversed for comparative purposes); (B) NHMUK R9140, *Zambiasaurus submersus* (left humerus). *Abbreviation*: *su*, supinator process. Scale bars equal 5 cm. [formatted for 2 column width].

The morphology of the humerus in *Eubrachiosaurus* differs strongly from that of *Placerias* (Figure 5A, 5D). The shape of the deltopectoral crest is similar in these two taxa, but this morphology is common among kannemeyeriiforms (also present in, e.g., *Ischigualastia*, *Stahleckeria*) (Figure 5B). *Placerias* also exhibits an unusual condition, otherwise known only in *Zambiasaurus* (Figure 6) and the unnamed Polish dicynodont [35]: a short ectepicondyle with a very tall, subvertical supinator process close to the shaft. In *Eubrachiosaurus*, the ectepicondyle is long, massive, and roughly rectangular, with an enlarged, nearly horizontal supinator process extending far out on the ectepicondyle. This morphology is closest to the condition in *Ischigualastia* (Figure 5B), as noted by Long and Murry [29], although similar ectepicondyles are also present in *Sinokannemeyeria* [30] and *Dinodontosaurus* [40]. *Stahleckeria* has a massive ectepicondyle as well, but with a more sloping dorsal edge and somewhat weaker supinator process [40]. In both *Eubrachiosaurus* and *Ischigualastia*, the rim of the trochlea is very pronounced dorsally, forming a prominent ridge beneath the attachment of the M. triceps humeralis medialis (Figures 5A, 5B). Development of this ridge (and size of the trochlea in general) is ontogenetically variable in kannemeyeriiforms (with weak development on the bone in juveniles, see Figures 5E, 5F). But even in specimens of similar size, this ridge is more weakly developed in *Stahleckeria* and *Placerias* (e.g., Figure 6A) than *Eubrachiosaurus* and *Ischigualastia*.

Although the humerus of *Eubrachiosaurus* is very similar to that of *Ischigualastia*, there are distinctions between them. The attachment site for the M. subcoracoscapularis is rounder and less pronounced in *Eubrachiosaurus* than *Ischigualastia*, and *Eubrachiosaurus* has a less acute angle at the proximal end of the deltopectoral crest (Figures 5A, 5B). The entepicondylar process of *Eubrachiosaurus* is larger and located more distally than in *Ischigualastia*. The humeral shaft of *Ischigualastia* is more massive than that of *Eubrachiosaurus*, although it is possible this is a preservational artifact in *Eubrachiosaurus*.

### Pelvis

**Figure 7:**
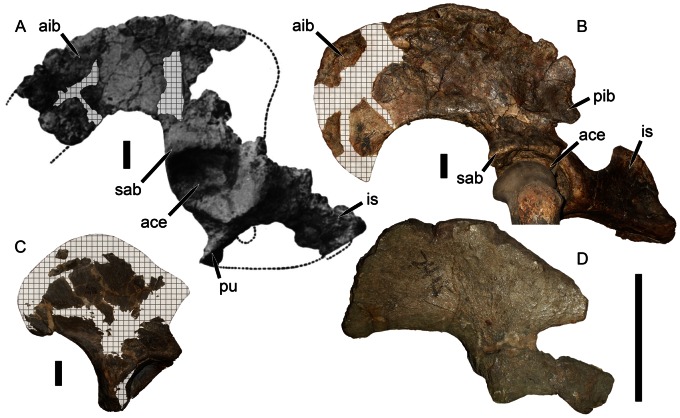
Kannemeyeriiform pelves in lateral view. (A) lost element from the holotype of *Eubrachiosaurus browni* (modified from [28]); (B) GPIT/RE/8001, *Stahleckeria potens* (acetabulum partially obscured by femur); (C) UCMP 32393, partial ilium of *Placerias hesternus*; (D) IVPP V2415, ilium and partial ischium of *Shansiodon wangi*. The specimens in A and D are left pelves, the specimens in B and C are right pelves that have been reversed for comparative purposes. Hatched areas indicate plaster reconstruction. Dashed lines in A show Williston’s [28] hypothesized shape of the complete pelvis. *Abbreviations*: *ace*, acetabulum; *aib*, anterior iliac blade; *is*, ischium; *pib*, posterior iliac blade; *pu*, pubis; *sab*, supraacetabular buttress. Scale bars equal 5 cm. [formatted for 2 column width].

**Figure 8:**
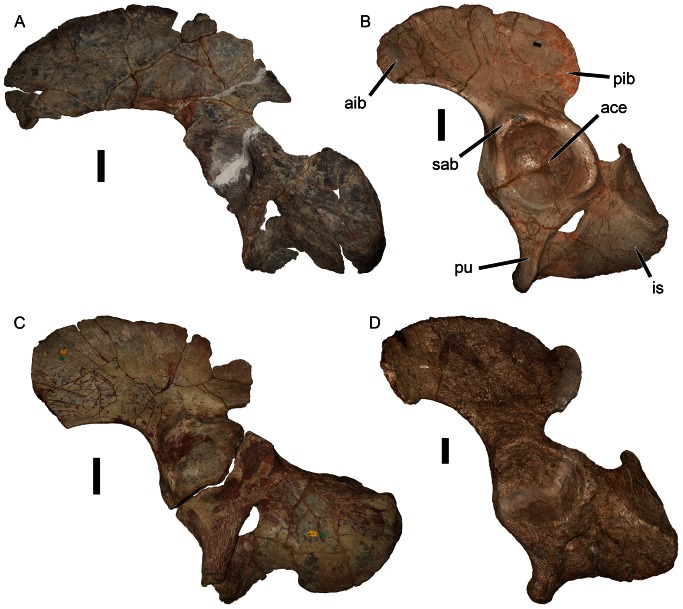
Kannemeyeriiform pelves in lateral view. (A) NHMUK R9732, *Angonisaurus cruickshanki*; (B) UFRGS PV-0150T, *Jachaleria candelariensis*; (C) NHMUK R3761, *Kannemeyeria simocephalus*; (D) PVL 3807, *Ischigualastia jenseni*. The specimen in A is a left pelvis, the specimens in B, C, and D are right pelves that have been reversed for comparative purposes. *Abbreviations*: *ace*, acetabulum; *aib*, anterior iliac blade; *is*, ischium; *pib*, posterior iliac blade; *pu*, pubis. Scale bars equal 5 cm. [formatted for 2 column width].

Williston’s description [28] indicates that the pelvis of *Eubrachiosaurus* was fragmentary, missing most of the posterior part of the ilium and the anterior tip of the iliac blade (also known as the anterior iliac process) (Figure 7A). The acetabulum was intact, with fragmentary antero- and posteroventral portions of the pubis and ischium (respectively). According to Williston [28], the dorsoventral width of the anterior iliac process was 17.0 cm, and the lengths of the pubis and ischium from the rim of the acetabulum were 14.5 cm and 20.0 cm (respectively). What was present of these bones was similar to the morphology seen in other kannemeyeriiforms (Figure 8). Kannemeyeriiforms generally have a long, anteroventrally curving anterior iliac process and short posterior iliac process. In some taxa the anteroventral curvature of the anterior iliac process is slight (e.g., *Shansiodon*, *Angonisaurus*) (Figures 7D, 8A). *Stahleckeria* has an extreme version of this morphology in which the anterior iliac process begins curving anteriorly immediately above the acetabulum and is particularly long (Figure 7B). More typical is the condition of *Kannemeyeria*, with a more weakly curved, shorter anterior process (Figure 8C). In *Ischigualastia*, the ilium is markedly constricted above the acetabulum (giving the appearance of an iliac ‘shaft’) and in this taxon and *Jachaleria* the anterior iliac blade is broadly rounded (Figures 8B, 8D). No unbroken iliac material is known for *Placerias*, but the most complete specimens (UCMP 25069, 32393) exhibit a morphology (Figure 7B) roughly similar to *Ischigualastia*, *Jachaleria*, and *Kannemeyeria*. *Eubrachiosaurus* had a distinctly elongate, strongly anteroventrally curving anterior iliac process (Figure 7A). Although incomplete, enough of this process was preserved to show that it had extreme downward curvature of the anteroventral margin of the blade above the acetabulum, differing from the typical *Kannemeyeria*-like condition. Williston’s [28] hypothetical outline of the pelvis of *Eubrachiosaurus* (dashed lines in Figure 7A) was made before kannemeyeriiform pelves were known from complete material and is probably inaccurate in several respects. In particular, it is likely that the pelvis of *Eubrachiosaurus*, like other kannemeyeriiforms, had a posterior iliac process.

### Phylogenetic Analysis

To determine the relationships of *Eubrachiosaurus browni*, we used a modified version of the anomodont matrix of Kammerer et al. [27]. Changes to that analysis (new taxa, new characters, and character state/coding revisions) are detailed below.

### Newly Included Taxa

The analysis of Kammerer et al. [27] used the most basal known anomodont, *Biseridens qilianicus* [41], as the outgroup. For this analysis we included non-anomodont outgroups, adding representatives of the other major therapsid groups: Biarmosuchia (represented by *Biarmosuchus tener* and *Hipposaurus boonstrai*), Dinocephalia (*Archaeosyodon praeventor* and *Titanophoneus potens*), Gorgonopsia (*Gorgonops torvus*), and Eutheriodontia (the basal therocephalians *Lycosuchus vanderrieti* and *Glanosuchus macrops*). These taxa were included to provide more robust polarity for anomodont characters, as *Biseridens* is known from fragmentary material and could not be coded for many characters (including all postcranial characters). It should be noted that although we added several characters to the analysis of Kammerer et al. [27] (see Appendix S1), our analysis remains focused on anomodont interrelationships, not global therapsid relationships, and we did not include many general therapsid characters (see, e.g., [42]).

We added four anomodont taxa to the analysis of Kammerer et al. [27]: the recently described basal anomodont *Tiarajudens eccentricus* from the Middle Permian of Brazil and three Triassic kannemeyeriiforms (*Eubrachiosaurus browni* from Wyoming, *Shaanbeikannemeyeria* from China, and *Zambiasaurus submersus* from Zambia). Two nominal species of *Shaanbeikannemeyeria* exist (*S*. *xilougouensis* Cheng, 1980 [43], the type, and *S*. *buerdongia* Li, 1980 [44]), but because they are extremely similar and probably synonymous we coded them as a single taxon here. Codings were based on personal examination of the type (for *Eubrachiosaurus* and *S*. *buerdongia*) and referred (for *Zambiasaurus*) specimens, examination of specimen casts (for the holotypes of *Z*. *submersus* and *T*. *eccentricus*), and reference to the literature (for *S*. *xilougouensis* and the lost elements of *Eubrachiosaurus* [28], [43]) (see Appendix S1 for specimen list).

### Excluded Kannemeyeriiform Taxa

Although we attempted to sample kannemeyeriiform dicynodonts as comprehensively as possible in the current analysis, some taxa necessarily required exclusion. We did not include a number of nominal Russian kannemeyeriiforms that are based on extremely fragmentary remains, several of which may represent *nomina dubia*. *Calleonasus furvus*, *Elatosaurus facetus*, and *Planitorostris pechoriensis* are known only from isolated nasals [45]–[47]. *Nasoplanites danilovi* and *Putillosaurus sennikovi* are known from premaxillary tips and partial palates [46], [48]. *Parvobestiola bashkiriensis* is known from a partial snout roof comprising the left nasal, prefrontal, and part of the frontal [46]. *Cristonasus koltaeviensis* is known from an isolated left fragment of premaxilla [46]. The diagnoses of these taxa rely heavily on bone surface ornamentation, which is problematic given that this feature varies both as a result of preservational artifact and ontogenetic change in kannemeyeriiforms (pers. obs. of *Dinodontosaurus pedroanum* and *Kannemeyeria simocephalus* growth series). The majority of these taxa (*Calleonasus*, *Cristonasus*, *Nasoplanites*, and *Parvobestiola*) are from the Middle Triassic Donguz Formation of Bashkortostan and their validity relative to each other and the more completely known Donguz kannemeyeriiforms (*Rabidosaurus*, *Rhadiodromus*, *Rhinodicynodon*, and *Uralokannemeyeria*) is suspect. Few of these taxa can be coded for any characters in our phylogenetic analysis. *Cristonasus* is notable for having the palatal surface of the premaxilla exposed in lateral view, a character state restricted to non-shansiodontid kannemeyeriiforms albeit variable within this group. Among Donguz taxa known from nearly complete skulls, this feature is only present in *Uralokannemeyeria*. Surkov [46] distinguished *Cristonasus* from *Uralokannemeyeria* based on relative snout width and premaxillary surface ornamentation, but this distinction is uncertain as the premaxilla is not completely preserved in either taxon. It is possible that these taxa are synonymous, but the holotype of *Cristonasus* is too incomplete to be sure.

*Edaxosaurus edentatus* was described based on an isolated left caniniform process (SGU D-104/4–1) from the Middle Triassic Donguz Formation of Orenburg [45]. Other than lacking a tusk, this caniniform process is very similar to that of *Uralokannemeyeria vjuschkovi* from the same locality. In particular, the strongly-developed ridge curving anterolaterally down the length of the caniniform process is unique to *Uralokannemeyeria* and the Indian *Rechnisaurus* among kannemeyeriiforms. Intraspecific tusk presence/absence is well known in Permian dicynodonts: in some taxa representing sexual dimorphism (e.g., *Diictodon*, *Pristerodon*) [49], [50] whereas in others it may represent individual variation (e.g., *Odontocyclops*, *Tropidostoma*, *Dicynodontoides*) [51]–[53]. Only tusked individuals are known for most dicynodontoids (the many known skulls of *Daptocephalus*, *Dicynodon*, *Kannemeyeria*, and *Lystrosaurus* are all tusked), indicating that both sexes were tusked in these taxa, but there are exceptions. Bandyopadhyay [31] described a tusked specimen of *Wadiasaurus indicus* (ISI R176), previously known from the tuskless holotype (ISI R38), arguing that these specimens represent the male and female of the species (respectively). Tusk development is also variable in *Placerias* [23]; they are present in some isolated maxillae (UCMP 27553), and absent in others (UCMP 137369). Given that the type specimens of *Edaxosaurus edentatus* and *Uralokannemeyeria vjuschkovi* are from the same locality, share several unique features among kannemeyeriiforms, and differ only in a character that is intraspecifically variable in at least some kannemeyeriiform species, we consider the most parsimonious interpretation to be that these specimens are conspecific, with *Edaxosaurus* representing a junior synonym of *Uralokannemeyeria*.

Of the Russian kannemeyeriiform ‘fragment taxa’, the only species that we recognize as clearly valid is *Elephantosaurus jachimovitschi*, known from a partial skull roof (PIN 525/25, preserving part of the frontals and the left prefrontal) and heavily worn tusks from the Middle Triassic Bukobay Formation of Bashkiria. This taxon has a unique prefrontal morphology: a large, smooth, rounded boss is present near the anterodorsal edge of the orbit and distinctly separated from a second, highly rugose prefrontal boss located anteriorly. *Elephantosaurus* is usually considered a stahleckeriid [15], [54], mostly on account of its large size (interorbital width estimated ∼20 cm), but the broad frontal contribution to the dorsal rim of the orbit in *Elephantosaurus* suggests that it falls outside of Stahleckeriidae. Although often described as an ‘enormous’ dicynodont, a very broad interorbital region is known in other kannemeyeriiforms: both *Sinokannemeyeria* and *Stahleckeria* exceed 20 cm interorbital width in some specimens, although the individual roofing bones in these taxa are not as thick as in *Elephantosaurus*. *Elephantosaurus* was certainly a large dicynodont, but its size cannot be securely estimated given the known material and may not have exceeded that of *Stahleckeria*. Although we consider *Elephantosaurus* valid, we did not include it in the current analysis, as it could only be coded for three characters in the data set.

With regards to taxa outside of Russia, we did not include *Azarifeneria*, known from two species (*A*. *barrati* and *A*. *robustus*) from the Triassic of Morocco [55], [56]. These species are based on extremely fragmentary, poorly-preserved material and their distinction from the sympatric *Moghreberia* is suspect. Finally, we excluded the gigantic dicynodont from the latest Triassic of Poland briefly described by Dzik et al. [35], as a complete description of this taxon is currently in progress (Sulej, pers. comm.).

### Character Additions and Emendations

We added one continuous character and ten discrete state characters to the analysis of Kammerer et al. [27]. Seven of the discrete state characters are phalangeal characters derived from previous analyses [42], three are new to this analysis. We emended a character concerning pterygoid dentition to address the unusual condition described for *Tiarajudens* and also recognized in *Anomocephalus* [57]. Finally, we recoded some character states for *Biseridens*, *Anomocephalus*, *Prosictodon, Syops*, *Rechnisaurus*, *Wadiasaurus*, *Uralokannemeyeria*, *Rhadiodromus*, *Stahleckeria*, and *Jachaleria* based on new examination of the material and fixed some typographic errors in the data matrix of Kammerer et al. [27]. Please refer to Appendix S1 for full details.

### Phylogenetic Methods

Our final data set includes 174 characters (see Appendices S2 and S3). One hundred fifty three of these characters are discrete binary or multistate characters, and we treated these characters as unordered and of equal weight. The remaining 21 characters are continuous. To code the continuous characters, we added a small number of new measurements to the database of Kammerer et al. [27]. Details of our measurement procedures and data processing for individual characters can be found in Kammerer et al. [27] and in Appendix S1, and the data matrix can be found as supporting information (S4). We treated the continuous characters as additive using the method of Goloboff et al. [58], and used mean values as the codings for the OTUs except in cases when only a single measurement was available for an OTU. We coded unknown and inapplicable discrete state and continuous characters as '?' [59].

We analyzed the data set using TNT 1.1 (October 2010 version) [60], and we employed two search strategies. For the first search, we used the new technology methods. We performed a driven search with the initial search level set at 65, which was checked every three hits. The initial number of addition sequence replicates was 5,000, and we required the search to find the trees of shortest length ten times. We started the analysis with default settings for tree fusing, tree drifting, parsimony ratchet, and sectorial searching. In the second analysis, we used the traditional search method of TBR branch swapping with 10,000 replicates, with 10 trees held per replicate. We used *Biarmosuchus* (see above) to root the most parsimonious cladograms from both analyses. To measure support for the most parsimonious cladograms, we utilized symmetric resampling [61] and decay analysis [62], [63]. Our symmetric resampling results are based on 15,000 replicates, with 10 replicates of TBR branch swapping with two trees held per replicate for each resampling replicate. The decay analysis results are based on a sample of 596,251 suboptimal cladograms with lengths up to seven steps longer than the most parsimonious cladograms. Following the recommendations of Goloboff et al. [60], we generated the suboptimal trees through a series of traditional searches in which we incrementally increased the length of suboptimal cladograms retained as well as the number of suboptimal cladograms. The resulting cladograms were filtered to remove duplicates before the decay analysis, so the 596,251 cladograms in the sample are all unique.

### Results of Phylogenetic Analysis

**Figure 9:**
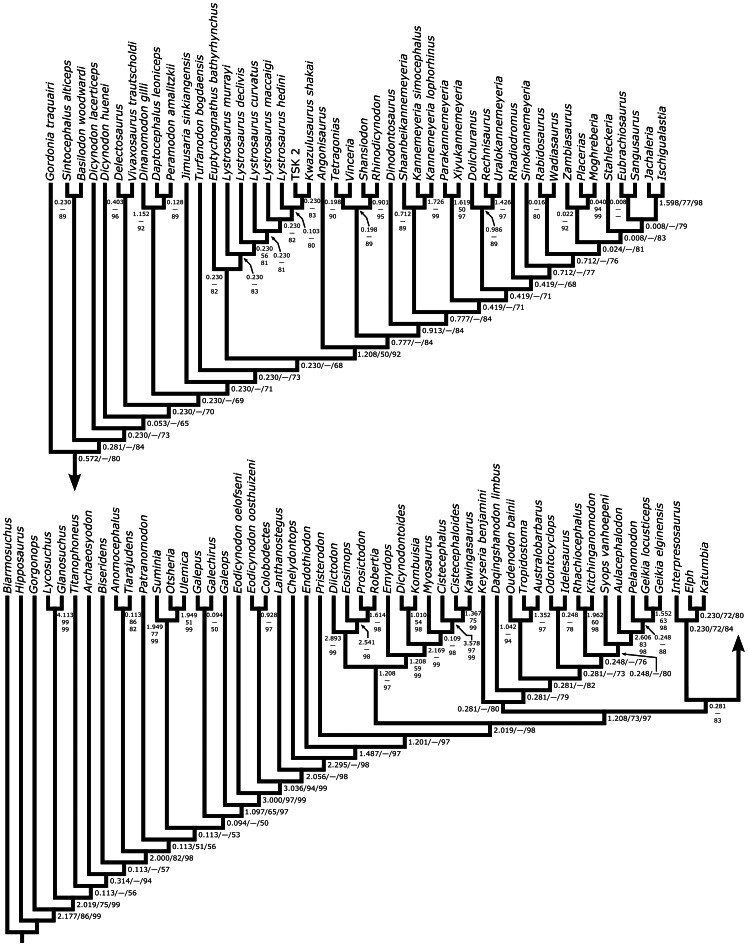
Most parsimonious cladogram from the phylogenetic analysis. Scores: 986.211 steps, consistency index = 0.244, retention index = 0.713. Numbers at nodes represent decay index (left/top), symmetric resampling (middle), and the percentage of the 596,251 suboptimal trees in which the node is resolved (right/bottom). [formatted for 2 column width].

Both the new technology searches and the traditional searches discovered the same most parsimonious cladogram (986.211 steps; CI = 0.244; RI = 0.713), and the topological results are summarized in Figure 9. Of particular importance in the current context is the fact that *Eubrachiosaurus* is reconstructed within the clade Stahleckeriinae (see below for the definition of this taxon) and not as a close relative of *Placerias*. There are also some differences between the current topology and that of Kammerer et al. [27], particularly in the arrangement of basal dicynodontoid species and among kannemeyeriiforms. In part, this likely stems from our inclusion of new characters and taxa in the analysis, as well as our revised coding for *Syops vanhoepeni*. At the same time, it is important to note that most nodes in Dicynodontoidea are weakly supported, and we expect there to be continued instability for some time as additional alpha taxonomic and phylogenetic work on this group proceeds.

Because this analysis is expanded from that of Kammerer et al. [27], it understandably agrees with it in most regards. In this section we will detail the novel results; for a more thorough description of all branches on the tree please refer to [27]. Relationships among the non-anomodont outgroups should not be taken as a serious phylogenetic hypothesis for these taxa, which were included to polarize characters within Anomodontia, not to analyze higher-level therapsid relationships. The topology outside of Anomodontia is an artifact of character choice, specifically our exclusion of most general therapsid characters and the characters most applicable to the outgroups.

The newly-added basal anomodont *Tiarajudens* was strongly supported as the sister taxon of *Anomocephalus*, as found by Cisneros et al. [57]. Outside of Dicynodontoidea, the only major difference between the current tree and that of Kammerer et al. [27] is the recovery of *Syops vanhoepeni* as a cryptodont rather than dicynodontoid. In the current topology this taxon is a geikiid, the sister taxon of Geikiinae (*Aulacephalodon*, *Pelanomodon*, and *Geikia*) sensu Kammerer and Angielczyk [64]. *Syops* was particularly volatile in our previous analyses, where it was coded based on the incomplete, poorly-prepared type specimens of *Dicynodon vanhoepeni* (SAM-PK-11311) and *D*. *roberti* (SAM-PK-11325A and B). The discovery of a new, more complete skull and mandible of *Syops* from the Luangwa Valley of Zambia (NHCC LB25 [65]) has permitted this taxon to be more thoroughly coded and clearly demonstrates its cryptodontian affinities. This material is currently under study by KDA and JF and will form the basis for a redescription of *S*. *vanhoepeni*.

Interrelationships within Dicynodontoidea varied strongly depending on the treatment of certain characters in the analyses of Kammerer et al. ([27]: compare Figs. 156, 157, and 159), and the majority of these nodes continue to be weakly supported in the present analysis. Notable features of the present topology include the recovery of “*Dicynodon*”-grade Karoo taxa with broad temporal bars (*Basilodon* and *Sintocephalus*) as basal dicynodontoids rather than lystrosaurids. Also close to the base of Dicynodontoidea, the Russian taxa *Vivaxosaurus* and *Delectosaurus* form a clade and *Dicynodon* (containing only *D*. *lacerticeps* and *D*. *huenei*) is paraphyletic, but these results are very weakly supported. A clade comprised of *Daptocephalus leoniceps*, *Dinanomodon gilli*, and *Peramodon amalitzkii* is recovered, as was the case in Kammerer et al. [27], but with a switch in the positions of *Daptocephalus* and *Dinanomodon*. The Chinese taxa *Jimusaria* and *Turfanodon* are found to lie outside of the Lystrosauridae-Kannemeyeriiformes split. The topology within Lystrosauridae remains problematic, with divergences opposite from the observed order of appearances in the stratigraphic record of *Lystrosaurus*. We believe these results are related to general instability in rooting within the Dicynodontoidea, and suggest that it may be instructive in future studies to compare the results of the current tree to an analysis focused on the Triassic dicynodontoid clades (Lystrosauridae and Kannemeyeriiformes).

Within Kannemeyeriiformes, the wildcard taxon *Angonisaurus* is recovered at the base of the clade. *Angonisaurus* exhibits a problematic mosaic of shansiodontid- and stahleckeriid-like characters [66], [67], and a very basal position for this taxon was recovered in some previous trees ([27]:Fig. 157). In the current analysis *Dinodontosaurus* lies outside of Shansiodontidae (here containing *Rhinodicynodon*, *Shansiodon*, *Tetragonias*, and *Vinceria*) as the sister taxon of the “kannemeyeriid” assemblage. “Kannemeyeriidae” is paraphyletic in the current topology, with an array of *Kannemeyeria*-grade taxa leading up to Stahleckeriidae. “Kannemeyeriid” paraphyly was recovered in some previous trees [27], but a notable change is the removal of *Wadiasaurus* and *Rhadiodromus* from Stahleckeriidae and *Dolichuranus* and *Rechnisaurus* from Shansiodontidae into “Kannemeyeriidae.” More stable is the recovery of a Stahleckeriidae with two major subclades, formalized below as Placeriinae (containing *Placerias*, *Moghreberia*, and *Zambiasaurus*) and Stahleckeriinae (containing *Stahleckeria*, *Ischigualastia*, *Jachaleria*, *Sangusaurus*, and *Eubrachiosaurus*).

## Discussion

### Validity of Eubrachiosaurus and Brachybrachium

*Eubrachiosaurus browni* is clearly distinct from *Placerias* on the basis of humeral and scapular morphology, most notably by its massive, rectangular humeral ectepicondyle with expanded supinator process. Although their humeri are similar, *Eubrachiosaurus* can be distinguished from *Ischigualastia* (as well as *Jachaleria*, for which the humerus is unknown) by their strikingly different iliac morphologies (rounded blade set off from acetabulum by shaft in *Ischigualastia*, elongate, anteroventrally curved blade in *Eubrachiosaurus*). The scapula of *Eubrachiosaurus* is similar to that of *Stahleckeria*, but narrower anteroposteriorly and more constricted at the level of the acromion. Scapular morphology also permits differentiation from *Placerias* (less expanded dorsally than *Eubrachiosaurus*) and *Ischigualastia*/*Jachaleria* (scapular spine absent, whereas it is well-developed in *Eubrachiosaurus*). This combination of characters, including the unique iliac curvature of *Eubrachiosaurus*, allows this taxon to be recognized as valid.

More problematic is *Brachybrachium brevipes*. Williston ([28]:p. 694) described this taxon on the basis of a fragmentary humerus from the upper Popo Agie beds, in “almost identically the same horizon” as *Eubrachiosaurus browni*. This specimen, now lost, was poorly preserved with much of the proximal and distal ends missing. It shares with the humerus of *Eubrachiosaurus* a nearly perpendicular angle between the proximal and distal edges of the deltopectoral crest. Williston [28] distinguished *Brachybrachium* from *Eubrachiosaurus* on the basis of a more massive trochlea and a more weakly-developed supinator process. However, the relative sizes of the distal processes on the humerus are intraspecifically variable in kannemeyeriiforms [23] and too little of the ectepicondyle was preserved in *Brachybrachium* to state with confidence that it differed appreciably from *Eubrachiosaurus*. Given that this specimen is from the same area and horizon as *E*. *browni* and exhibits no clear morphological differences from that taxon, it is likely that these two taxa are synonymous. However, on a strict apomorphy basis *Brachybrachium brevipes* must be considered a *nomen dubium*.

### Kannemeyeriiform Higher-level Taxonomy

Kammerer and Angielczyk [64] proposed a higher-level taxonomy for Permian anomodonts, but did not do the same for Triassic dicynodonts because of the lack of comprehensive phylogenetic analyses up to that point. Previous phylogenetic analyses of kannemeyeriiform taxa [26], [68]–[70] produced conflicting topologies and included only a fraction of kannemeyeriiform diversity. Although the current analysis includes almost all valid kannemeyeriiform taxa, relationships within the clade remain weakly supported, and it is likely that they will continue to change in future studies. As such, a formalized taxonomy of most kannemeyeriiform subclades remains premature. This said, there are a few clades that have been consistently recovered in the analyses of Kammerer et al. [27] and the current study that should be formally recognized for ease of communication.

Kannemeyeriiformes Maisch, 2001 [26] was established to refer to the large clade of Triassic non-lystrosaurid dicynodontoids, breaking from taxonomies that treated this group as a subfamily of Dicynodontidae [8]. The monophyly of Kannemeyeriiformes has been recovered in all subsequent analyses [27], [70]: inclusion of “*Dicynodon*”-grade taxa in phylogenetic analyses demonstrates that these taxa lie outside of Kannemeyeriiformes, rather than representing basal members of the kannemeyeriiform subclades. Here we define Kannemeyeriiformes as *Kannemeyeria simocephalus* (Weithofer, 1888) [71] and all taxa more closely related to it than to *Lystrosaurus murrayi* (Huxley, 1859) [72] or *Dicynodon lacerticeps* Owen, 1845 [73]. We use a stem-based definition for this taxon as counterpart to its sister taxon Lystrosauridae. Although no Permian kannemeyeriiforms are currently known, this clade must extend back to at least the Late Permian *Cistecephalus* Assemblage Zone, when the first lystrosaurids appear [27]. The lengthy pre-Anisian ghost lineage of kannemeyeriiforms is one of the major mysteries of dicynodont evolution: kannemeyeriiforms are large, robust animals with a high preservation potential, suggesting that their absence in well-sampled Late Permian basins (e.g., Karoo Basin of South Africa, Russian fore-Urals) is a biogeographic issue rather than within-basin taphonomic bias. Kannemeyeriiformes is supported by the following synapomorphies in our analysis: (1) absence of the postfrontal bone on the dorsal surface of the skull (discrete state character 39:state 1); (2) postorbitals do not extend for entire length of intertemporal bar, posterior portion of bar formed only by parietals (reversal of this character is common in Kannemeyeriiformes, however) (49∶1); (3) distinct dorsolateral notch in squamosal below zygomatic arch in posterior view absent (54∶0); (4) intertuberal ridge absent (90∶0); (5) lateral dentary shelf present but relatively small (110∶1); (6) anterolateral trough for the posterior process of the dentary absent on angular (116∶0); (7) procoracoid does not participate in formation of glenoid (130∶0); and (8) insertion of M. subcoracoscapularis on humerus a short, pinna-like process (132∶2).

Within kannemeyeriiforms, many authors have recognized variations of Shansiodontidae, Kannemeyeriidae, and Stahleckeriidae as the primary subgroups [24], and we agree that these names correspond to common kannemeyeriiform morphotypes (see, e.g., discussion of kannemeyeriiform scapulae above). The monophyly of the first two groups is suspect, however. A group of “core shansiodontids” has been recovered in all of our analyses, but the shansiodontid-like taxa *Angonisaurus* and *Dinodontosaurus* behave as wildcards. More problematic still is the recovery of *Kannemeyeria*-like taxa (“kannemeyeriids”) in varying degrees of paraphyly relative to Stahleckeriidae ([27] and the current analysis). Only Stahleckeriidae has retained relatively stable composition across our analyses (with the exception of occasional inclusion of *Wadiasaurus* and *Rhadiodromus*). As defined here, Stahleckeriidae comprises the last common ancestor of *Placerias hesternus* Lucas, 1904 [74] and *Stahleckeria potens* Huene, 1935 [40] and all of its descendents, so long as this group does not include *Shansiodon wangi* Yeh, 1959 [75] or *Kannemeyeria simocephalus* (Weithofer, 1888) [71] (Note: Kammerer et al. [27] used the spelling *Kannemeyeria simocephala* for this taxon, following its wide usage in the therapsid literature. However, because this specific epithet was erected as a noun in apposition, it is undeclinable, and the original spelling *simocephalus* must be maintained.) Stahleckeriidae is supported by one unambiguous synapomorphy in our analysis: (1) interparietal making a large contribution to the skull roof (52∶2).

Stahleckeriidae contains two subfamilies with stem-based definitions to form a node-stem triplet. Placeriinae (King, 1988) [8] comprises all taxa more closely related to *Placerias hesternus* Lucas, 1904 [74] than to *Stahleckeria potens* Huene, 1935 [40]. This clade contains *Placerias*, *Moghreberia*, and *Zambiasaurus*. Placeriinae is supported by the following synapomorphies in our analysis: (1) distinct lateral caniniform buttress absent (27∶0); and (2) origin of triceps on posterior surface of scapula developed into a distinct posterior projection (147∶1). Stahleckeriinae Lehman, 1961 [39] comprises all taxa more closely related to *Stahleckeria potens* Huene, 1935 [40] than to *Placerias hesternus* Lucas, 1904 [74]. This clade contains *Stahleckeria*, *Eubrachiosaurus*, *Ischigualastia*, *Jachaleria*, and *Sangusaurus*. Stahleckeriinae is supported by the following synapomorphies in our analysis: (1) palatal surface of premaxilla exposed in lateral view (19∶1); (2) frontal contribution to the dorsal rim of the orbit thin or absent (38∶1); and (3) four sternal bosses (124∶1).

### Other Non-*Placerias* Dicynodont Material from the Triassic of Western North America

**Figure 10:**
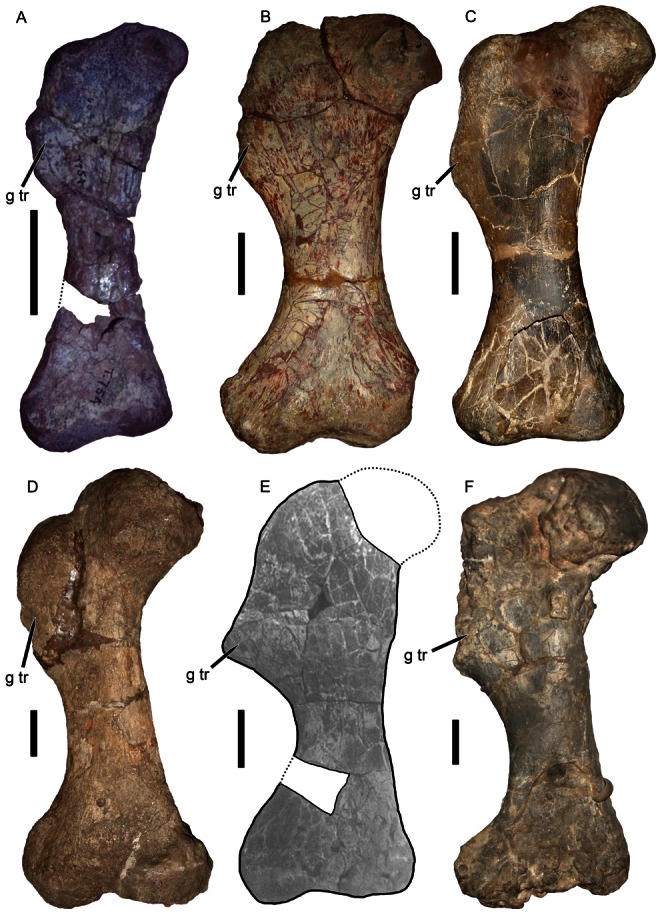
Kannemeyeriiform femora in anterior view. (A) CAMZM T754, *Tetragonias njalilus*; (B) NHMUK R3740, *Kannemeyeria simocephalus*; (C) UCMP 32394, *Placerias hesternus*; (D) PVL 3807, *Ischigualastia jenseni*; (E) NMMNH P-13001 (modified from [17]); (F) GPIT/RE/8001, *Stahleckeria potens*. Dotted lines indicate unpreserved areas. The specimens in A and B are right femora, the specimens in C, D, E, and F are left femora that have been reversed for comparative purposes. *Abbreviation*: *g tr*, greater trochanter. Scale bars equal 5 cm. [formatted for 2 column width].

Lucas and Hunt [17] described several postcranial elements of a large dicynodont from the Los Esteros Member of the Santa Rosa Formation (Santa Fe County, New Mexico). They identified this material as cf. *Ischigualastia* sp., based primarily on the morphology of a nearly-complete left femur (NMMNH P-13001). They correctly noted that the short, stout femoral shaft and prominent greater trochanter of this specimen preclude identification as *Placerias* (10C). However, this femur (Figure 10E) also differs from that of *Ischigualastia*, particularly in the morphology of the greater trochanter: it is highly flared, with an acute angle between the lateral and ventral margins, unlike the low, rounded condition in *Ischigualastia* (Figure 10D). Among kannemeyeriiforms, this type of sharply-angled greater trochanter is also observed in *Stahleckeria* (Figure 10F); in most other taxa the trochanter is relatively low (Figures 10A–D). It should be noted, however, that the greater trochanter in NMMNH P-13001 is even more widely flared than that of *Stahleckeria* and also lacks the straight, vertical lateral margin of that taxon, instead curving outwards.

NMMNH P-13001 appears to represent a taxon of kannemeyeriiform dicynodont that is distinct from *Placerias* and is similar to *Stahleckeria*. Given that the only known Late Triassic stahleckeriine in western North America is *Eubrachiosaurus* (based on the phylogenetic analysis above), it is possible that the New Mexican specimen represents another individual of *Eubrachiosaurus browni*. Unfortunately, the paucity of overlapping material between these two individuals makes it impossible at present to state whether they are conspecific. The New Mexican specimen includes a scapular fragment (NMMNH P-13003), but it is too incomplete to permit meaningful comparisons with the scapula of FMNH UC 633. The other elements of the New Mexican specimen (NMMNH P-13002, an incomplete radius; NMMNH P-13004, a phalanx; and NMMNH P-13005, an incomplete axis vertebra) preserve only the general kannemeyeriiform morphology and do not aid in lower-level identification. The discovery of more complete specimens of *Eubrachiosaurus* preserving the femur will be necessary to resolve the status of the New Mexican specimen, and it should be considered Stahleckeriidae indet. for the time being. Other than the New Mexican record, the only other western North American dicynodont material that is not referable to *Placerias* is a record from the Dockum Group of Texas [76], but this material has yet to be properly described and its possible relationships are currently unknown.

### Kannemeyeriiform Material from Eastern North America

**Figure 11:**
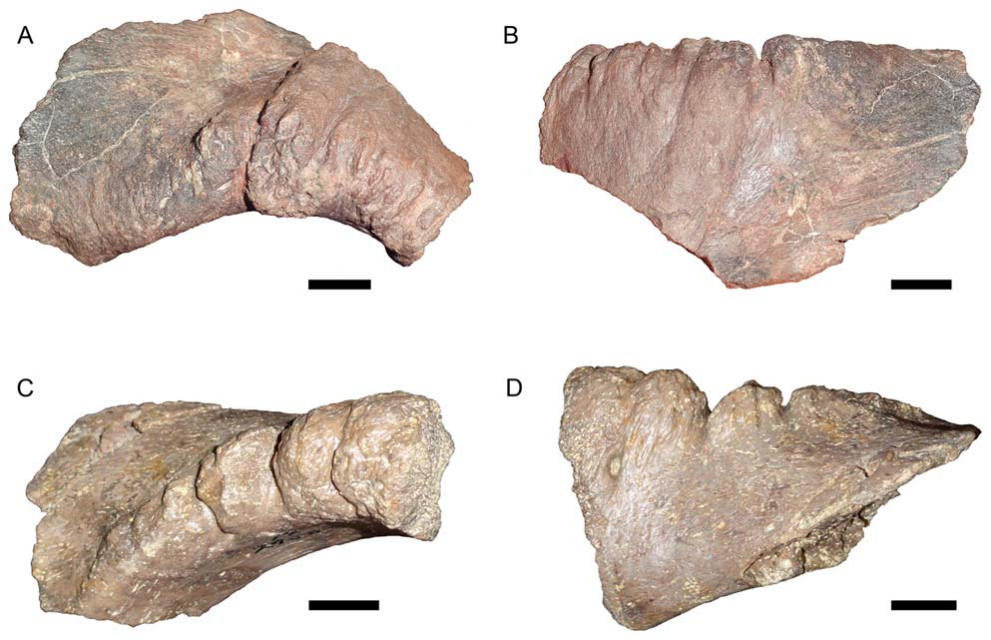
Postorbitals of *Placerias hesternus* showing characteristic rugosity. AMNH FARB 2851, from the Pekin Formation of North Carolina, in lateral (A) and posterior (B) views; MNA V2950, from the Chinle Formation of Arizona, in lateral (C) and posterior (D) views. Scale bars equal 1 cm. [formatted for 2 column width].

Kannemeyeriiform fossils are known from several localities in the Late Triassic Newark Supergroup of eastern North America. The first named North American dicynodont, *Dicynodon rosmarus*, was described based on an isolated tooth and tooth root from Phoenixville, Pennsylvania (New Oxford Formation, Newark Supergroup) [77]. However, these teeth (now lost) may have been from a phytosaur rather than a dicynodont [27]. The first definitive dicynodont specimens from the eastern U.S. were described by Baird and Patterson [21] from the Pomona Pipe Products Pit, Pekin Formation of North Carolina. This material was referred to *Placerias* [22], operating under the idea that this taxon is the only dicynodont from the Late Triassic of North America. With the revalidation of *Eubrachiosaurus*, we have demonstrated that this is not the case. However, our reexamination of the Pomona material reveals autapomorphies that confirm identification as *Placerias hesternus*. The postorbital bones of *Placerias* exhibit a distinct pattern of rugose ornamentation, unique among kannemeyeriiforms, in which a series of deep furrows and grooves are present (Figure 11). Rugose circumorbital ornamentation is typical of many kannemeyeriiform species, and highly rugose postorbitals are also known in *Jachaleria candelariensis* (UFRGS PV0151T), but no other taxon (including *Jachaleria*) has the extremely deep furrows present in *Placerias* (Figures 11B, 11D).

Green et al. [78] reported on new dicynodont material from the Upper Triassic Pekin Formation in North Carolina that seems to be distinct from *Placerias*. The material comprises a partial articulated postcranial skeleton (NCSM 21719), including the posterior dorsal and sacral regions of the vertebral column, and nearly complete pelvic girdle and hind limbs. Green [79] figured NCSM 21719, which has an anteroventrally-curved anterior iliac blade. The preserved femur, however, does not have a sharp, expanded greater trochanter as in NMMNH P-13001. This suggests that at least two non-*Placerias* stahleckeriids are present in the Late Triassic of North America. More complete material is required to determine which (if either) of the North Carolina and New Mexico specimens is referable to *Eubrachiosaurus*, however.

### Kannemeyeriiform Diversity and Distribution in the Late Triassic

The recognition of *Eubrachiosaurus* as a valid kannemeyeriiform taxon belonging to a distinct lineage (Stahleckeriinae) from *Placerias* alters our understanding of the Late Triassic fossil record of dicynodonts. Although their lower abundance and absolute richness compared to Middle Triassic faunas do attest to a clade in decline, the concept of Norian kannemeyeriiforms as highly geographically-restricted relicts is no longer tenable. Instead, this is the time of highest diversity and geographic range among one of the major kannemeyeriiform subclades, Stahleckeriidae, and among stahleckeriids there is evidence of continued dispersal and diversification in both recognized subfamilies. Stahleckeriines are first known from the African Middle Triassic (the Anisian *Sangusaurus* from Tanzania and Zambia) but include three taxa in the Late Triassic of South America and one (*Eubrachiosaurus*) in North America in what is most parsimoniously interpreted as trans-Pangaean dispersal. Placeriines also include a taxon from the African Middle Triassic (*Zambiasaurus* from the Anisian of Zambia) but are best known from the Norian of North America (*Placerias*) and Morocco (*Moghreberia*), as well as potentially including the giant Rhaetian taxon from Poland [35]. The presence of two non-*Placerias* femoral morphologies in the North American Late Triassic record (see above) suggest that at least one additional stahleckeriid other than *Eubrachiosaurus* and *Placerias* was present. Combined with other recent discoveries [80], these records suggest that large kannemeyeriiforms were a component of American and European faunas throughout the Late Triassic. Intriguingly, kannemeyeriiforms occur in several regions where traversodontid cynodonts are absent (e.g., western North America), complicating interpretations of synapsids as humid-belt-restricted taxa [2]. Although progressive Triassic aridification remains the primary hypothesis for synapsid decline, it is clear that arid regions were at least tolerated by stahleckeriid dicynodonts, and further research into the paleobiology and physiology of these massive herbivores is necessary.

## Supporting Information

Appendix S1 **Additions and emendations to the phylogenetic analysis.** Details changes made between the previously published version of this data set [27] and the current analysis.
(DOC)

Appendix S2 **List of continuous characters used in the phylogenetic analysis.**
(DOC)

Appendix S3 **List of discrete state characters used in the phylogenetic analysis.**
(DOC)

Appendix S4 **Data matrix used in the phylogenetic analysis.**
(NEX)
