# Supplementary material for: On the Validity and Phylogenetic Position of Eubrachiosaurus browni, a Kannemeyeriiform Dicynodont (Anomodontia) from Triassic North America
Source: PLoS One. 2013 May 31;8(5):e64203. doi: 10.1371/journal.pone.0064203 (PMC3669350; doi:10.1371/journal.pone.0064203)
Supplement: Appendix S1 — Additions and emendations to the phylogenetic analysis. Details changes made between the previously published version of this data set [27] and the current analysis. (DOC) [file pone.0064203.s001.doc]

APPENDIX S1

**Newly added characters**

Character numbering has been changed from that of Kammerer et al. (2011) in order to keep continuous and discrete state character lists separate (see Appendices S2 and S3) and improve ease of coding. The 174 characters in the analysis are now broken into continuous characters (CC) 1-21 and discrete state characters (DC) 1-153. The following characters are new to the analysis:

Continuous

21. Breadth of scapula measured as ratio of maximal proximal width of scapula versus length of scapula (measured from dorsal edge of glenoid to proximal tip).

Discrete state

38. Frontal contribution to the dorsal rim of the orbit: broad, frontal forms a major part of the orbital rim (0); thin or absent, if present a thin frontal process extends laterally between the prefrontal and postorbital to reach the orbital margin (1). In most anomodonts, the frontal forms a significant portion if not the majority of the dorsal margin of the orbit. In *Ischigualastia*, *Jachaleria*, *Sangusaurus*, and *Stahleckeria*, however, enlargement of the prefrontal limits frontal contribution to the orbital margin. In *Ischigualastia*, *Sangusaurus*, and *Stahleckeria*, only a thin frontal process extending laterally between the prefrontal and the postorbital reaches the orbital margin. In *Jachaleria*, the frontal is completely excluded from the orbital margin. The only other anomodont without a frontal contribution to the orbital rim is *Cistecephaloides*.

47. Parietals bulge outwards as ovoid swellings at posterior end of sagittal crest: no (0); yes (1). *Wadiasaurus* is characterized by extremely inflated parietals at the posterior end of the sagittal crest, with the parietals diverging posteriorly as two swollen, ovoid structures in dorsal view. Although this extreme morphology is autapomorphic for *Wadiasaurus*, the same morphology is developed to a lesser degree in *Dolichuranus* and *Kannemeyeria*, with a ‘lip’-like swollen divergence between the postorbitals posteriorly that is not observed in other kannemeyeriiforms.

76. Ectopterygoid dentition absent (0) or present (1). Ectopterygoid dentition in anomodonts is only known in *Anomocephalus* and *Tiarajudens*, which have the unusual morphology of a labiolingually expanded postcanine tooth row borne on the ectopterygoid and pterygoid instead of the maxilla.

139. Manual digit III, shape of second phalanx: long (0), short (disc-like) (1), absent (2). (From Sidor and Hopson, 1998: 152)

140. Manual digit IV, phalangeal number: 5 (0), 4 (1), or 3 (2). (From Sidor and Hopson, 1998: 153)

141. Manual digit IV, shape of second and third phalanges: long (0), short (1). (From Sidor and Hopson, 1998: 154)

149. Pedal digit III, shape of second phalanx: long (0), short (disc-like) (1), absent (2). (From Sidor and Hopson, 1998: 178)

150. Pedal digit IV, phalangeal number: 5 (0), 4 (1), or 3 (2). (From Sidor and Hopson, 1998: 179)

151. Pedal digit IV, shape of second and third phalanges: long (0), short (1). (From Sidor and Hopson, 1998: 180)

152. Pedal digit V, shape of second phalanx: long (0), short (1), absent (2). (From Sidor and Hopson, 1998: 181)

**Emended characters**

DC 82 altered from Kammerer et al. (2011) (was character 88), now: Pterygoid dentition: present, conical (0); absent (1); present, bucco-lingually expanded (2). (incorporating Character 75 of Cisneros et al., 2011)

**Taxon codings changed from Kammerer et al. (2011)**

The codings for continuous characters 2 and 9 were transposed in the data matrix of Kammerer et al. (2011), this has been corrected in the current matrix.

*Biseridens* coded for DC 19 (palatal surface of premaxilla not exposed in lateral view, 0) based on IVPP V16013.

*Anomocephalus* has been recoded for several characters following reinterpretation of its cranial morphology by comparison to *Tiarajudens*: DC 18, maxillary alveolar region length, has been changed from 1, long, to 0, short; DC 21, maxillary non-caniniform teeth, has been changed from 0 (located near lateral margin of maxilla) to 2 (absent); DC 22 and 23, which have to do with the morphology of maxillary non-caniniform teeth, have been changed to “?” as these teeth are not present in *Anomocephalus*; DC 82, pterygoid dentition, changed from ? to 2 (present, bucco-lingually expanded).

*Prosictodon* coded for DC 8 (posterior process of premaxilla with a bifurcated posterior tip, 1); DC 27 (distinct lateral caniniform buttress absent, 0); DC 31 (nasals with a long median suture that separates the premaxilla from the frontals, 0); DC 50 (fossa on the ventral surface of the intertemporal bar formed by the postorbital and parietal large, 0); and DC 51 (pineal foramen surrounded by a strong, rugose boss, 2); recoded for DC 20, maxillary canine, changed from 1 (absent) to 2 (present as tusk); and DC 33, naso-frontal suture, changed from 0 (relatively straight) to 1 (with a distinct anterior process) based on new referred specimen, BP/1/7190.

*Dicynodontoides* recoded for DC 43 (preparietal morphology) changed from 4 (typographical error, state absent) to 1 (flat and flush with the skull roof).

*Idelesaurus* recoded for DC 45 (parietal posterolateral process) changed from 2 (typographical error, state absent) to 1 (short).

*Syops* coded for DC 4 (posterior median ridge on palatal surface of premaxilla present with a flattened, expanded anterior area, 1); DC 5 (palatal surface of premaxilla relatively flat with no depressions present, 2); DC 6 (premaxillary teeth absent, 2); DC 9 (palatine shelf ventral to internal naris, 1); DC 35 (lacrimal contacts septomaxilla, 1); DC 45 (parietal posterolateral process short, 1); DC 52 (interparietal does not contribute to intertemporal skull roof, 0); DC 55 (squamosal posteroventral process long such that nearly all of the quadrate and quadratojugal are covered by the squamosal in posterior view, 1); DC 60 (sutural contact of squamosal and maxilla present, 1); DC 61 (squamosal contacts supraoccipital, 1); DC 63 (quadrate with a dorsal lobe that has a convex, rounded anterior edge that rests against quadrate ramus of pterygoid, 0); DC 64 (vomers fused, 1); DC 65 (mid-ventral plate of vomers without a notable expanded area posterior to junction with premaxilla, 1); DC 66 (mid-ventral plate of vomers narrow and blade-like in ventral view, 1); DC 67 (trough on mid-ventral plate of vomers absent, 1); DC 68 (palatine dentition absent, 1); DC 69 (palatal surface of palatine highly rugose and textured, suggesting a keratinized covering, with a raised posterior section and an anterior section that is smoother and flush with the secondary palate, 2); DC 70 (palatine widens anteriorly forming a palatine pad, 2); DC 71 (foramen on the palatal surface of the palatine absent, 0); DC 72 (lateral palatal foramen present at the level of the anterior, expanded palatal exposure of the palatines, 1); DC 73 (sutural contact of palatine and premaxilla present, 1); DC 74 (labial fossa surrounded by maxilla, jugal, and palatine present, 1); DC 75 (ectopterygoid does not extend further posteriorly than palatine in palatal aspect, 1); DC 79 (contact of pterygoid and maxilla present, 1); DC 82 (pterygoid dentition absent, 1); DC 88 (stapedial facet of basisphenoid-basioccipital tuber exposed ventrolaterally and open distally, 2); DC 91 (dorsal process on anterior end of epipterygoid footplate absent, 0); DC 94 (tabular separated from opisthotic by squamosal, 1); DC 95 (exoccipital and basioccipital contributions to the occipital condyle distinct, 0); DC 96 (occipital condyle distinctly tri-radiate in posterior view, 1); DC 97 (circular central depression on the occipital condyle between the exoccipitals and basioccipital present, 0); DC 99 (mandibular fenestra present, 1); DC 117 (reflected lamina of angular widely separated from articular, 1); DC 119 (articular at least partially fused to prearticular, 1); DC 120 (surangular vertical lamina absent,1); and DC 121 (jaw articulation permits parasagittal movement, 1) based on new referred specimen NHCC LB25.

*Dinanomodon* recoded for DC 117 (proximity of reflected lamina of angular to articular) changed from 2 (typographical error, state absent) to 1 (widely separated).

*Rechnisaurus* recoded for DC 19 (palatal surface of premaxilla exposed in lateral view), changed from 0 (absent) to 1 (present); DC 43 (preparietal bone), changed from 1 (present and flush with skull roof) to 2 (present and in a depression); DC 46 (parietal exposure) changed from 2 (narrow and crest-like) to 1 (exposed in midline groove) based on reexamination of the holotype ISI R37.

*Wadiasaurus* recoded for DC 16 (narial notch) from 0 (absent) to 1 (present) based on reexamination of the holotype ISI R38. DC 20 (maxillary tusk) changed from 1 (absent) to [1/2] (polymorphic) based on referred specimen ISI R176.

*Uralokannemeyeria* recoded for DC 46 (parietal exposure) changed from 2 (narrow and crest-like) to 1 (exposed in midline groove).

*Rhadiodromus* recoded for DC 20 (maxillary tusk) from 1 (absent) to 2 (present as tusk) based on condition in PIN 1579/14.

*Stahleckeria* recoded for DC 16 (narial notch) from 0 (absent) to 1 (present) based on condition in the lectotype GPIT/RE/8000.

*Jachaleria* recoded for DC 16 (narial notch) from 0 (absent) to 1 (present) based on condition in UFRGS PV0147T.

**Specimens examined to code new taxa**

The specimens used to code the data matrix are the same as in Kammerer et al. (2011), with emendations of previously-included taxa noted above. For the eleven new taxa added to the analysis, codings were based on firsthand examination of the following specimens: *Biarmosuchus tener* (PIN 1758/1, 1758/2, 1758/7, 1758/8, 1758/18, 1758/19, 1758/255); *Hipposaurus boonstrai* (SAM-PK-8950); *Archaeosyodon praeventor* (PIN 1758/3, 1758/93, 1758/95, 1758/293, 1758/294, 1758/297, 1758/309, 1758/314, 1748/328); *Titanophoneus potens* (PIN 157/1, 157/3); *Gorgonops torvus* (AMNH FARB 5515, NHMUK R1647); *Lycosuchus vanderrieti* (CGP 60, FMNH UC 1707, US D173); *Glanosuchus macrops* (SAM-PK-637, 11843, K7808, K7809); *Tiarajudens eccentricus* (cast of UFRGS PV393P); *Eubrachiosaurus browni* (FMNH UC 633); *Shaanbeikannemeyeria* *buerdongia* (IVPP V6033); and *Zambiasaurus submersus* (NHMUK R9000, 9001, 9002, 9003, 9005, 9011, 9013, 9016, 9020, 9021, 9026, 9029, 9033, 9034, 9036, 9039, 9040, 9047, 9068, 9069, 9073, 9080, 9086, 9089, 9091, 9096, 9098, 9103, 9106, 9109, 9113, 9118, 9122, 9123, 9128, 9140).

**References**

Cisneros JC, Abdala F, Rubidge BS, Dentzien-Dias PC, Bueno A (2011) Dental occlusion in a 260-million-year-old therapsid with saber canines from the Permian of Brazil. Science 331: 1603-1605.

Kammerer CF, Angielczyk KD, Fröbisch J (2011) A comprehensive taxonomic revision of *Dicynodon* (Therapsida, Anomodontia) and its implications for dicynodont phylogeny, biogeography, and biostratigraphy. Soc Vertebr Paleontol Mem 11: 1-158.

Sidor CA, Hopson JA (1998) Ghost lineages and ‘mammal-ness’: assessing the temporal pattern of character acquisition in the Synapsida. Paleobiol 24: 254-273.
