# Supplementary material for: On the Validity and Phylogenetic Position of Eubrachiosaurus browni, a Kannemeyeriiform Dicynodont (Anomodontia) from Triassic North America
Source: PLoS One. 2013 May 31;8(5):e64203. doi: 10.1371/journal.pone.0064203 (PMC3669350; doi:10.1371/journal.pone.0064203)
Supplement: Appendix S2 — List of continuous characters used in the phylogenetic analysis. (DOC) [file pone.0064203.s002.doc]

# APPENDIX S2

# Continuous (morphometric) characters

1. Length of preorbital region of skull relative to basal length of skull. (Modified from Modesto et al., 1999: 6) (=Kammerer et al., 2011 [K]: 1)
2. Relative length of premaxillary secondary palate (From Angielczyk 2007: 63) (=K2)
3. Minimum width of interorbital skull roof relative to basal length of skull. (=K3)
4. Relative width of temporal bar at level of postorbital bar versus the relative width at the junction of the intertemporal bar with the occipital plate. (=K4)
5. Length of temporal fenestra relative to basal length of skull. (=K5)
6. Width of median pterygoid plate relative to basal skull length. (=K6)
7. Angle formed by the posterior pterygoid rami. (=K7)
8. Length of interpterygoid vacuity relative to basal length of skull. (Modified from Angielczyk and Kurkin, 2003: 33) (=K8)
9. Relative area of the internal nares. (From Angielczyk, 2007: 64) (=K9)
10. Angle between ascending and zygomatic processes of the squamosal. (Modified from Fröbisch and Reisz 2008: 75) (=K10)
11. Angulation of the occiput relative to the palate, expressed the ratio of dorsal and basal lengths of the skull. (Modified from Cox and Li 1983) (=K11)
12. Ratio of length to height of mandibular fenestra in lateral view. (Modified from Rubidge and Hopson, 1996: 5; and Modesto et al., 1999: 36) (=K12)
13. Ratio of height of dentary ramus to height of dentary symphysis. (=K13)
14. Ratio of maximum height of postdentary bones (excluding reflected lamina of angular) to the height of the dentary ramus. (=K14)
15. Ratio of minimum width of the scapula to maximum width of dorsal end of scapula. (From Angielczyk, 2007: 72) (=K15)
16. Length of the deltopectoral crest relative to total length of the humerus. (From Angielczyk, 2007: 68) (=K16)
17. Maximum width of the distal end of the radius relative to the maximum length of the radius. (From Angielczyk, 2007: 69) (=K17)
18. Ratio of posterior iliac process length to acetabulum diameter. (From Sidor and Hopson, 1998: 157) (=K18)
19. Ratio of anterior iliac process Length to acetabulum diameter. (From Sidor and Hopson, 1998: 158) (=K19)
20. Length of trochanteric crest on femur relative to length of femur. (=K20)
21. Breadth of scapula measured as ration of maximal proximal width of scapula versus length of scapula (measured from dorsal edge of glenoid to proximal tip). (new)

**References**

Angielczk KD (2007) New specimens of the Tanzanian dicynodont “*Cryptocynodon*” *parringtoni* von Huene, 1943 (Therapsida, Anomodontia), with an expanded analysis of Permian dicynodont phylogeny. J Vertebr Paleontol 27: 116-131.

Angielczyk KD, Kurkin AA (2003) Phylogenetic analysis of Russian Permian dicynodonts (Therapsida: Anomodontia): implications for Permian biostratigraphy and Pangaean biogeography. Zool J Linn Soc 139: 157-212.

Cox CB, Li J (1983) A new genus of Triassic dicynodont from east Africa and its classification. Palaeontol 26: 389-406.

Fröbisch J, Reisz RR (2008) A new species of *Emydops* (Synapsida, Anomodontia) and a discussion of dental variability and pathology in dicynodonts. J Vertebr Paleontol 28: 770-787.

Kammerer CF, Angielczyk KD, Fröbisch J (2011) A comprehensive taxonomic revision of *Dicynodon* (Therapsida, Anomodontia) and its implications for dicynodont phylogeny, biogeography, and biostratigraphy. Soc Vertebr Paleontol Mem 11: 1-158.

Modesto S, Rubidge B, Welman J (1999) The most basal anomodont therapsid and the primacy of Gondwana in the evolution of the anomodonts. Proc Roy Soc London B 266: 331-337.

Rubidge BS, Hopson JA (1996) A primitive anomodont therapsid from the base of the Beaufort Group (Upper Permian) of South Africa. Zool J Linn Soc 117: 115-139.

Sidor CA, Hopson JA (1998) Ghost lineages and ‘mammal-ness’: assessing the temporal pattern of character acquisition in the Synapsida. Paleobiol 24: 254-273.
