# Supplementary material for: On the Validity and Phylogenetic Position of Eubrachiosaurus browni, a Kannemeyeriiform Dicynodont (Anomodontia) from Triassic North America
Source: PLoS One. 2013 May 31;8(5):e64203. doi: 10.1371/journal.pone.0064203 (PMC3669350; doi:10.1371/journal.pone.0064203)
Supplement: Appendix S3 — List of discrete state characters used in the phylogenetic analysis. (DOC) [file pone.0064203.s003.doc]

# APPENDIX S3

# Discrete state characters

1. Premaxillae unfused (0) or fused (1). (From Angielczyk and Kurkin, 2003: 3) (=Kammerer et al., 2011 [K]: 21)
2. Paired anterior ridges on palatal surface of premaxilla absent (0), present and converge posteriorly (1), or present and do not converge (2). (From Angielczyk and Kurkin, 2003: 7) (=K22)
3. Lateral anterior palatal ridges absent (0) or present (1). (From Angielczyk, 2007: 60) (=K23)
4. Posterior median ridge on palatal surface of premaxilla absent (0), present with a flattened, expanded anterior area (1), or present without a flattened, expanded anterior area (2). (From Angielczyk and Kurkin, 2003: 8) (=K24)
5. Palatal surface of premaxilla with well-defined depressions with curved sides lateral to median ridge (if present) (0), with groove-like depressions that have straight sides and a rounded anterior end (1), or relatively flat with poorly defined or no depressions present (2). (From Angielczyk and Kurkin, 2003: 9) (=K25)
6. Location of premaxillary teeth lateral (0), medial (1) or absent (2). (From Angielczyk and Kurkin, 2003: 2) (=K26)
7. Posterior exposure of the premaxilla on the palate: absent (0), present (1). (From Hopson and Barghusen, 1986: 6.8) (=K27)
8. Posterior process of the premaxilla with a non-bifurcated posterior tip (0) or with a bifurcated posterior tip (1). (=K153)
9. Palatine shelf ventral to internal naris: absent (0), present (1). (From Hopson and Barghusen, 1986: 20.3 and 21.4) (=K28)
10. Anterior tip of snout rounded (0) or squared off (1). (From Maisch and Gebauer, 2005: 11) (=K29)
11. Marked anterior expansion of preorbital region absent (0) or present (1). (Modified from Maisch and Gebauer, 2005: 2) (=K30)
12. Snout roughly parallel to long axis of skull (0) or strongly angled ventrally (1). (=K31)
13. Snout dorsoventrally shallow (0) or dorsoventrally deepened (1). (=K156)
14. Snout open to back of the skull (0) or anterior margin of orbit extended posteromedially to partly close off the snout from the rest of the skull (1). (From Angielczyk and Kurkin, 2003: 25) (=K32)
15. Septomaxilla posterodorsal spur present and widely separates nasal and maxilla (0), spur present but does not separate maxilla and nasal (i.e., nasal-maxilla suture present and well defined in this region) (1), septomaxilla spur absent (2). (Modified from Modesto et al., 1999: 8) (=K33)
16. Notch on dorsal edge of narial opening absent (0) or present (1). (From Kammerer et al., 2011: 34) (=K34)
17. Postnarial excavation absent (0), present, relatively small, and rounded posteriorly (1), or present, very large, and elongate (2). (Vega-Dias et al, 2004: 8) (=K35)
18. Maxillary alveolar region short, occupying less than 53% of the ventral length of the bone (0) or tooth bearing region long, occupying 72% or more of the ventral length of the bone (1). (From Modesto et al., 1999: 9) (=K36)
19. Palatal surface of premaxilla exposed in lateral view (1) or not exposed in lateral view (0). (=K37)
20. Maxillary canine present as large member of tooth series (0), absent (1), or present as tusk (2). (From Modesto et al., 2003: 6) (=K38)
21. Maxillary non-caniniform teeth located near lateral margin of maxilla (0), located more medially, (1), or absent (2). (From Angielczyk and Kurkin, 2003: 4) (=K39)
22. Shelf-like area lateral to the maxillary non-caniniform teeth absent (0) or present (1). (From Angielczyk and Kurkin, 2003: 5) (=K40)
23. Fine serrations on maxillary teeth present (0), serrations absent (1), or coarse serrations present (2). (From Modesto et al., 1999: 3) (=K41)
24. Sutural contact of maxilla and prefrontal present (0) or absent (1). (From Modesto et al., 2003: 10) (=K42)
25. Caniniform process absent (0) or present (1). (Modified from Angielczyk and Kurkin, 2003: 6) (=K43)
26. Caniniform depression: has the form of an embayment of palatal rim anterior to caniniform process or tusk (1), has the form of a notch in palatal rim anterior to caninfiorm process (2), or absent (0). (Modified from Angielczyk and Kurkin, 2003: 6 and 14) (=K44)
27. Distinct lateral caniniform buttress absent (0), present (1), or present with posteroventral furrow (2). (From Damiani et al., 2007: 25) (=K152)
28. Keel-like extension of the palatal rim posterior to the caniniform process absent (0) or present (1). (From Angielczyk and Kurkin, 2003: 1) (=K45)
29. Postcaniniform crest absent (0) or present (1). (From Angielczyk and Kurkin, 2003: 28) (=K46)
30. Ventral edge of the caniniform process or dorsal edge of the erupted portion of the canine tusk anterior (0) to, or at the same level to slightly posterior to (1) the anterior orbital margin. (From Angielczyk and Kurkin, 2003: 44) (=K47)
31. Nasals with a long median suture that separates the premaxilla from the frontals (0) or with a short median suture and frontals and premaxilla in close proximity (1) (Modified from Vega-Dias et al., 2004: 4) (=K155)
32. Nasal bosses absent (0), present as a median swelling with a continuous posterior margin (1), present as paired swellings near the dorsal or posterodorsal margin of external nares (2), present as paired swellings that meet in the midline to form a swollen anterodorsal surface on the snout (3). (Modified from Angielczyk and Kurkin, 2003: 23) (=K48)
33. Naso-frontal suture relatively straight, interdigitated, or gently bowed (0), with a distinct anterior process (1), or with a distinct posterior process (2). (Modified from Vega-Dias et al., 2004: 7) (=K154)
34. Transverse crest approximately at level of naso-frontal suture absent (0) or present (1). (From Maisch, 2002: 18) (=K49)
35. Lacrimal does not contact septomaxilla (0) or does contact septomaxilla (1). (From Vega-Dias et al., 2004: 9). (=K151)
36. Prefrontal bosses absent (0), present but separate from nasals (1), or present and confluent with nasal bosses (2). (=K50)
37. Raised, sometimes rugose, circumorbital rim absent (0) or present (1). (=K51)
38. Frontal contribution to the dorsal rim of the orbit: broad, frontal forms a major part of the orbital rim (0); thin or absent, if present a thin frontal process extends laterally between the prefrontal and postorbital to reach the orbital margin (1). (new)
39. Postfrontal bone present on dorsal surface of skull (0) or absent (1). (From Maisch, 2002: 8) (=K52)
40. Postorbital bar without (0) or with thickenings and rugosities (1). (From Maisch and Gebauer, 2005: 5) (=K53)
41. Mediolateral flattening and anteroposterior expansion of postorbital bar for most or all of its length absent (0) or present (1). (=K143)
42. Temporal portion of skull roof relatively straight, without a strong break in slope (0), or temporal portion of skull roof angled dorsally with a strong break in slope near its anterior end (1). (Modified from Vega-Dias et al. 2004: #16) (=K54)
43. Preparietal bone absent (0), present and its dorsal surface relatively flat and flush with the skull roof (1), present and with a depressed dorsal surface relative to the surrounding skull roof (2), present with ridges running along it which may surround the pineal foramen (3). (From Angielczyk and Kurkin, 2003: 45) (=K55)
44. Parietals’ contribution to skull table transversely as broad as long (0), longer anteroposteriorly than broad (1), or shorter anteroposteriorly than broad (2). (From Modesto and Rybczynski, 2000: 16) (=K56)
45. Parietal posterolateral process slender and elongate (0), or short (1). (From Modesto and Rybczynski, 2000: 17) (=K57)
46. Parietals well exposed on the skull roof and relatively flat (0), parietals exposed in midline groove or channel (1), dorsal parietal exposure narrow and crest-like (2). (Modified from Kammerer et al., 2011: 58) (=K58)
47. Parietals bulge outwards as ovoid swellings at posterior end of sagittal crest: no (0); yes (1). (new)
48. Orientation of the temporal portion of the postorbital: relatively flat, so that most of the exterior surface of the bone faces dorsally (0), close to vertical, so that most of the exterior surface of the bone faces laterally (1), or bi-planar, with approximately equally-sized dorsal and lateral surfaces that are close to perpendicular (2). (=K59)
49. Postorbitals extend the entire length of intertemporal bar (0) or do not extend the entire length of intertemporal bar, such that the posterior portion of the bar is formed only by the parietals (1). (Modified from Maisch, 2001: 1; Vega-Dias et al., 2004: 13) (=K146)
50. Fossa on the ventral surface of the intertemporal bar formed by the postorbital and parietal large (0), reduced (1), or absent (2). (From Angielczyk and Kurkin, 2003: 53) (=K60)
51. Pineal foramen surrounded by a thin, smooth, chimney-like boss (0), flush or nearly flush with dorsal surface of skull (1), surrounded by a strong, often rugose boss (2), surrounded by a boss but with pineal canal angled anteriorly (3), or absent (4). (Modified from Angielczyk and Kurkin, 2003: 26) (=K61)
52. Interparietal does not contribute to intertemporal skull roof (0), makes a small contribution to intertemporal skull roof (1), or makes a large contribution to intertemporal skull roof (2). (Modified from Angielczyk and Kurkin, 2003: 48; and Maisch 2001: 2; Vega-Dias et al., 2004: 15) (=K62)
53. Squamosal without (0) or with (1) lateral fossa for the origin of the lateral branch of the M. adductor mandibulae externus. (From Angielczyk and Kurkin, 2003: 21) (=K63)
54. Distinct dorsolateral notch in squamosal below zygomatic arch in posterior view absent (0) or present (1). (Modified from Angielczyk and Kurkin, 2003: 32) (=K64)
55. Squamosal posteroventral process short such that there is relatively extensive exposure of quadrate and quadratojugal in posterior view and the quadrate foramen (if present) is visible in posterior view) (0) or long such that nearly all of the quadrate and quadratojugal are covered by the squamosal in posterior view and the quadrate foramen (if present) is not visible in posterior view (1). (Modified from Modesto et al., 1999: 15) (=K65)
56. Zygomatic portion of the squamosal of nearly constant thickness (0), with posterior portion thickened and/or downturned (1), or with thin dorsoventral expansion posterior to postorbital bar (2). (Modified from Angielczyk and Kurkin, 2003: 51) (=K66)
57. Zygomatic process of squamosal parasagittally deep (0), narrow and rod-like (1), or transversely expanded (2). (From Modesto et al., 1999: 12) (=K67)
58. Oblique ridge on lateral side of zygomatic arch giving triangular cross-section and overhanging a weak groove present (1) or absent (0). (=K157)
59. Squamosal zygomatic process narrowly based and in line with occipital condyle (0) or widely based and flares posteriorly beyond occipital condyle (1). (From Modesto et al., 2003: 15) (=K68)
60. Sutural contact of squamosal and maxilla absent (0) or present (1). (From Angielczyk and Kurkin, 2003: 34) (=K69)
61. Squamosal separated by tabular bone from supraoccipital (0) or contacts supraoccipital (1). (From Modesto et al., 1999: 20) (=K70)
62. Quadratojugal narrow and rod-like (0) or plate-like distally (1). (From Modesto et al., 1999: 17) (=K71)
63. Quadrate with a dorsal lobe that has a convex, rounded anterior edge that rests against quadrate ramus of pterygoid (0) or with a dorsal lobe that is developed into a distinct process that extends anteriorly along the quadrate ramus of the pterygoid and is triangular to sub-triangular in shape (1). (=K72)
64. Vomers unfused (0) or fused (1). (From Angielczyk and Kurkin, 2003: 11) (=K73)
65. Mid-ventral plate of vomers with an expanded, oval-shaped area posterior to junction with premaxilla (0) or without a notable expanded area posterior to junction with premaxilla (1). (From Angielczyk and Kurkin, 2003: 12) (=K74)
66. Mid-ventral plate of vomers relatively wide in ventral view (0), more narrow and blade-like in ventral view (1). (Modified from Angielczyk and Kurkin, 2003: 13) (=K75)
67. Trough on mid-ventral plate of vomers (i.e., ventral surface concave ventrally with raised edges): present (0) or absent (1). (=K76)
68. Palatine dentition present (0) or absent (1). (From Modesto et al., 1999: 25) (=K77)
69. Palatal surface of the palatine without evidence of a keratinized covering (0), relatively smooth but with fine pitting and texturing suggestive of a keratinized covering (1), highly rugose and textured, suggesting a keratinized covering, with a raised posterior section and an anterior section that is smoother and flush with the secondary palate (2), moderately rugose with pitting suggesting a keratinized covering for all of surface and flush with the secondary palate (3), smooth and unornamented medially but with very rugose, foveate lateral margin at contact with maxilla (4). (Modified from Angielczyk and Kurkin, 2003: 22) (=K78)
70. Palatine widest at its approximate midpoint of length (0), widens posteriorly (1), widens anteriorly forming a palatine pad (2), or width relatively constant for entire length (3). (Modified from Rybczynski, 2000: 23; and Angielczyk, 2004: 30) (=K79)
71. Foramen on the palatal surface of the palatine absent (0) or present (1). (From Angielczyk and Kurkin, 2003: 24) (=K80)
72. Lateral palatal foramen absent (0), present at level of the anterior, expanded palatal exposure of the palatines (1), present posterior and dorsal to the level of the anterior, expanded palatal exposure of the palatines (2). (From Angielczyk and Kurkin, 2003: 35) (=K81)
73. Sutural contact of palatine and premaxilla absent (0) or present (1). (From Angielczyk and Kurkin, 2003: 27) (=K82)
74. Labial fossa surrounded by maxilla, jugal, and palatine absent (0) or present (1). (From Angielczyk, 2001: 19; Angielczyk and Kurkin 2003: 19) (=K163)
75. Ectopterygoid extends further posteriorly than palatine in palatal aspect (0), or does not extend further posteriorly than palatine in palatal aspect (1), or absent (2). (Modified from Modesto et al., 1999: 24) (=K83)
76. Ectopterygoid dentition absent (0) or present (1). (new)
77. Pterygoids contact anteriorly (0) or separated by vomers (1). (Modified from Modesto et al., 1999: 29) (=K84)
78. Transverse flange of pterygoid projects laterally, free of posterior ramus (0), projects laterally, bound by posterior ramus (1), ventrally directed and relatively large, wing-like (2), or ventrally directed and low, keel-like (3). (Modified from Modesto et al., 2003: 31; and Rybczynski, 2000: 27) (=K85)
79. Contact of pterygoid and maxilla absent (0) or present (1). (From Angielczyk and Kurkin, 2003: 46) (=K86)
80. Converging ventral keels on posterior portion of anterior pterygoid rami absent (0) or present (1). (=K150)
81. Ventral surface of the median pterygoid plate depressed (0), smooth and flat (1), with a thin median ridge (2), with a wide, boss-like median ridge (3), or with a low rugose median swelling (4), or with a conical ventral projection (5). (Modified from Angielczyk and Kurkin, 2003: 43) (=K87)
82. Pterygoid dentition present, conical (0); absent (1); present, bucco-lingually expanded. (Modified from Modesto et al., 1999: 26; Cisneros et al., 2011: 75) (=K88)
83. Posterior edges of the interpterygoid vacuity located dorsal to the median pterygoid plate (0) or extended ventrally such that they are flush with the median pterygoid plate (1).(=K89)
84. Development of the pila antotica as a rod-like process on the anterior edge of the periotic with a corresponding notch for the trigeminal never posterior to it (0), or pronounced pila antotica absent and trigeminal notch is a horizontal hollow in the anterior edge of the periotic (1). (From Surkov and Benton 2004: 12) (=K90)
85. Contact between periotic and parietal absent (0) or present (1). (From: Surkov and Benton 2004: 15) (=K91)
86. Parasphenoid excluded from (0) or reaches (1) interpterygoid vacuity. (From Modesto et al., 1999: 32) (=K92)
87. Basisphenoid contribution to the basisphenoid-basioccpital tubera slopes anterodorsally at a shallow angle, forming elongate ridges on the basicranium that are close to the same height as the tubera for most of their length (0), slopes anterodorsally at a steeper angle such that the parabasisphenoid conribution is still somewhat ridge-like but the portion of the ridge on the anterior surface of the tuber is more vertically-oriented (1), or is nearly vertical, forming very weak ridges if any (2). (From Angielczyk and Rubidge, 2013: 77) (=K93)
88. Stapedial facet of basisphenoid-basioccipital tuber exposed laterally (0), exposed ventrolaterally (1), or exposed ventrolaterally and open distally (2). (Modified from Angielczyk and Kurkin, 2003: 42) (=K94)
89. Margin of fenestra ovalis formed predominantly by parabasisphenoid, with little or no contribution from basioccipital (0), formed by approximately equal portions of parabasisphenoid and basioccipital (1), or formed predominantly by basioccipital, with little or no contribution by parabasisphenoid (2). (From Angielczyk, 2007: 54) (=K95)
90. Intertuberal ridge absent (0) or present (1). (From Angielczyk and Kurkin, 2003: 49) (=K96)
91. Dorsal process on anterior end of epipterygoid footplate absent (0) or present (1). (From Angielczyk and Rubidge 2010: 73) (=K97)
92. Stapedial foramen present (0) or absent (1). (From Angielczyk and Kurkin, 2003: 29) (=K98)
93. Dorsal process of the stapes present (0) or absent (1). (From Fröbisch, 2007: 72) (=K99)
94. Tabular contacts opisthotic (0) or separated from opisthotic by squamosal (1). (From Modesto et al., 1999: 21) (=K100)
95. Exoccipital and basioccipital contributions to the occipital condyle distinct (0) or co-ossified into a single unit (1). (=K101)
96. Occipital condyle round to subspherical in posterior view (0) or distinctly tri-radiate (1) in posterior view. (=K144)
97. Circular central depression or fossa on the occipital condyle between the exoccipitals and basioccipital present (0) or absent (1). (=K147)
98. Floccular fossa present (0) or absent (1). (From Angielczyk and Kurkin, 2003: 41) (=K102)
99. Mandibular fenestra absent (0), present (1), or present but occluded by a thin sheet of the dentary (2). (Modified from Rubidge and Hopson, 1996: 5, and Modesto et al., 1999: 36) (=K103)
100. Jaw ramus straight in dorsal view, without strong lateral bends (0), or bends strongly laterally (1) posterior to symphysis. (=K104)
101. Dentaries sutured (0) or fused (1) at symphysis. (From Modesto et al., 1999: 33) (=K105)
102. Teeth present on dorsal surface of dentaries (0), medially displaced, sometimes on a swelling or shelf (1), or absent (2). (From Angielczyk and Kurkin, 2003: 10) (=K106)
103. Fine serrations on dentary teeth present (0), serrations absent (1), or coarse serrations present (2). (Modified rom Modesto et al., 1999: 3) (=K107)
104. Denticulated cingulum on dentary teeth absent (0) or present (1). (Modified from Modesto et al., 1999: 4) (=K108)
105. Symphyseal region of lower jaw smoothly rounded and at same level as rest of dentary ramus in lateral view (0), with an upturned margin that is raised above the level of the dorsal surface of the jaw rami and has a scooped-out depression on its posterior surface (1), drawn into a sharp, spiky beak (2), or shovel-shaped with a rounded or squared-off edge and a weak depression on its posterior surface (3). (From Angielczyk and Kurkin, 2003: 18) (=K109)
106. Curved ridge that follows the profile of the symphysis present on the edge between the anterior and lateral surfaces of the dentary absent (0) or present (1). (new) (=K142)
107. Dentary table absent (0) or present (1). (From Angielczyk and Rubidge, 2013: 15) (=K110)
108. Posterior dentary sulcus absent (0), present but does not extend past dentary teeth (if present) (1), present and extends past dentary teeth (if present), but is relatively wide and shallow (2), or present, extends past dentary teeth (if present) and is narrower and deeper (3). (From Angielczyk and Rubidge, 2013: 16) (=K111)
109. Tall, dorsally-convex cutting blade on medial edge of dorsal surface of dentary absent (0) or present (1). (From Angielczyk and Rubidge, 2013: 78). (=K112)
110. Lateral dentary shelf absent (0), present but relatively small (1), present and well developed (2). (Modified from Fröbisch and Reisz 2008: 82) (=K113)
111. Anterodorsal edge of lateral dentary shelf relatively flat (0), with a groove (1), or developed into a rounded swelling (2). (=K114)
112. Lateral dentary shelf relatively thick, with distinct dorsal and ventral surfaces above the mandibular fenestra (0) or a thin ventrolaterally-directed sheet that forms the dorsal margin of the mandibular fenestra (1). (=K148)
113. Splenial symphysis unfused (0) or fused (1). (From Sidor, 2001: 26) (=K115)
114. Spenial contribution to dentary symphysis: anterior process on splenial present in ventral view (0) or absent (1). (=K116)
115. Coronoid bone present (0), or absent (1). (From Modesto et al., 1999: 38) (=K117)
116. Angular with anterolateral trough for the posterior process of the dentary absent (0) or present (1) (=K118)
117. Reflected lamina of angular closely approaches or touches articular (0) or widely separated from articular (1). (From Maisch, 2001: 28) (=K158)
118. Prearticular with (0) or without (1) lateral exposure posteriorly. (From Modesto et al., 1999: 39) (=K119)
119. Articular distinct (0) or at least partially fused to prearticular (1). (From Sidor, 2001: 48) (=K120)
120. Surangular vertical lamina present and lateral to articular (0) or absent (1). (From Modesto et al., 1999: 37) (=K121)
121. Jaw articulation permits strictly orthal closure (0) or permits parasagittal movement (1). (From Rybczynski, 2000: 37; and Angielczyk, 2004: 41) (=K122)
122. Enlarged dentary caniniform present (0) or absent (1). (=K123)
123. Number of sacral vertebrae three (0), four (1), five (2), or six (3). (From Angielczyk and Kurkin, 2003: 36) (=K124)
124. Number of sternal bosses: 2 (0), 4(1). (From Vega-Dias et al., 2004: 32) (=K161)
125. Cleithrum absent (0) or present (1). (From Angielczyk and Kurkin, 2003: 39) (=K125)
126. Anterior edge of scapula extended laterally to form a strong crest (1) or not (0). (Modified from Vega-Dias et al., 2004: 28) (=K159)
127. Origin of triceps on posterior surface of scapula relatively low (0) or developed into a prominent posterior projection (1). (Modified from Vega-Dias et al., 2004: 26) (=K160)
128. Acromion process: absent or very small (0) or present and well defined (1). (Modified from Sidor and Hopson, 1998: 134; and Vega-Dias et al., 2004: 27) (=K126)
129. Procoracoid foramen or notch entirely contained within the procoracoid (0) or formed by contributions of the procoracoid and scapula in lateral view (1). (From Angielczyk, 2007: 66) (=K127)
130. Procoracoid does not participate in formation of glenoid (0) or participates in formation of glenoid (1). (From Angielczyk, 2007: 67) (=K128)
131. Proximal articular surface of humerus formed by a slightly convex area on proximal surface of the bone without much expansion onto the dorsal surface (0), somewhat expanded with some encroachment onto the dorsal surface (1), or strongly developed and set off from rest of humerus by a weak neck (2). (From Angielczyk and Kurkin, 2003: 30) (=K129)
132. Insertion of M. subcoracoscapularis on humerus a rounded, rugose area on proximal end of humerus (0), large elongate process (1), or short, pinna-like process (2). (Modified from Angielczyk, 2007: 58) (=K130)
133. Insertion of M. latissimus dorsi at rugose tuberosity on the posteroventral surface of humerus (0) or extended into a dorsoventrally flattened pinna-like process (1). (From Angielczyk and Kurkin, 2003: 50) (=K131)
134. Anterior and distal edges of deltopectoral crest close to perpendicular (0) or very obtuse (1) (Modified from Govender and Yates 2009: 78). (=K149)
135. Ectepicondylar foramen on humerus present (0) or absent (1). (From Angielczyk and Kurkin, 2003: 38) (=K132)
136. Radial and ulnar condyle continuous (0) or well ossified and separate (1) on ventral surface of humerus. (From Surkov et al., 2005: 12) (=K133)
137. Ulna with small olecranon process that does not extend far past the articular surface for the humerus (0), or with a large olecranon process that extends well past the articular surface for the humerus (1). (Angielczyk, 2007: 61) (=K135)
138. Distal carpal 5: present as a distinct element (0), not present as a distinct element (1). (Modified from Sidor and Hopson, 1998: 151) (=K140)
139. Manual digit III, shape of second phalanx: long (0), short (disc-like) (1), absent (2). (From Sidor & Hopson, 1998: 152)
140. Manual digit IV, phalangeal number: 5 (0), 4 (1), or 3 (2). (From Sidor & Hopson, 1998: 153)
141. Manual digit IV, shape of second and third phalanges: long (0), short (1). (From Sidor & Hopson, 1998: 154)
142. Dorsal edge of ilium: unnotched (0) or notched (1). (=K134)
143. Pubic plate is significantly expanded anteroposteriorly, such that its length is comparable to that of ischium (0) or anteroposteriorly short, so that it is much shorter than ischium (1). (=K136)
144. Pubic plate is significantly expanded ventrally such that it is nearly the same height as ischium (0) or reduced ventrally such that it is shorter than ischium (1). (Modified from Surkov et al., 2005: 14) (=K145)
145. Distinct cranial process on anterior end of pubis absent (0) or present (1). (=K137)
146. Femoral head continuous with the dorsal margin of femur (0) or offset dorsally from dorsal margin (1). (Modified from Govender and Yates, 2009: 84) (=K162)
147. Proximal articular surface of the femur present as a weak swelling that is mostly limited to the proximal surface of the bone (0) or present as a more rounded, hemispherical swelling that has some encroachment on the anterior surface of the femur (1). (Modified from Angielczyk and Kurkin, 2003: 31) (=K138)
148. Insertion of M. iliofemoralis present as a low rugosity on the dorsolateral portion of the femur (0), developed into a distinct crest that extends down part of the lateral surface of the femur (1) or a lateral crest that is split into a distinct first trochanter and third trochanter (2). (Modified from Angielczyk and Kurkin, 2003: 52; and Govender and Yates, 2009: 85) (=K139)
149. Pedal digit III, shape of second phalanx: long (0), short (disc-like) (1), absent (2). (From Sidor & Hopson, 1998: 178)
150. Pedal digit IV, phalangeal number: 5 (0), 4 (1), or 3 (2). (From Sidor & Hopson, 1998: 179)
151. Pedal digit IV, shape of second and third phalanges: long (0), short (1). (From Sidor & Hopson, 1998: 180)
152. Pedal digit V, shape of second phalanx: long (0), short (1), absent (2). (From Sidor & Hopson, 1998: 181)
153. Greatly enlarged vascular channels present (1) or absent (0). (From Angielczyk, 2007: 74) (=K141)

**References**

Angielczyk KD (2001) Preliminary phylogenetic analysis and stratigraphic congruence of the dicynodont anomodonts (Synapsida: Therapsida). Palaeontol Afr 37: 53-79.

Angielczyk KD (2004) Phylogenetic evidence for and implications of a dual origin of propaliny in anomodont therapsids. Paleobiol 30: 268-296.

Angielczk KD (2007) New specimens of the Tanzanian dicynodont “*Cryptocynodon*” *parringtoni* von Huene, 1943 (Therapsida, Anomodontia), with an expanded analysis of Permian dicynodont phylogeny. J Vertebr Paleontol 27: 116-131.

Angielczyk KD, Kurkin AA (2003) Phylogenetic analysis of Russian Permian dicynodonts (Therapsida: Anomodontia): implications for Permian biostratigraphy and Pangaean biogeography. Zool J Linn Soc 139: 157-212.

Angielczyk KD, Rubidge BS (2010) A new pylaecephalid dicynodont (Therapsida, Anomodontia) from the *Tapinocephalus*  Assemblage Zone, Karoo Basin, Middle Permian of South Africa. J Vertebra Paleontol 30: 1396-1409.

Angielczyk KD, Rubidge BS (2013) Skeletal morphology, phylogenetic relationships, and stratigraphic range of *Eosimops newtoni* Broom, 1921, a pylaecephalid dicynodont (Therapsida, Anomodontia) from the Middle Permian of South Africa. J Sys Palaeontol 11: 191-231.

Cisneros JC, Abdala F, Rubidge BS, Dentzien-Dias PC, Bueno A (2011) Dental occlusion in a 260-million-year-old therapsid with saber canines from the Permian of Brazil. Science 331: 1603-1605.

Cox CB, Li J (1983) A new genus of Triassic dicynodont from east Africa and its classification. Palaeontol 26: 389-406.

Damiani R, Vasconcelos C, Renaut A, Hancox J, Yates A (2007) *Dolichuranus primaevus* (Therapsida: Anomodontia) from the Middle Triassic of Namibia and its phylogenetic relationships. Palaeontol 50: 1531-1546.

Fröbisch J, Reisz RR (2008) A new species of *Emydops* (Synapsida, Anomodontia) and a discussion of dental variability and pathology in dicynodonts. J Vertebr Paleontol 28: 770-787.

Govender R, Yates A (2009) Dicynodont postcrania from the Triassic of Namibia and their implications for the systematics of kannemyeriiforme dicynodonts. Palaeontol Afr 44: 41-57.

Hopson JA, Barghusen HR (1986) An analysis of therapsid relationships. In: Hotton N III, MacLean PD, Roth JJ, Roth EC, editors. The ecology and biology of mammal-like reptiles. Washington, DC: Smithsonian Institution Press. pp. 83-106.

Kammerer CF, Angielczyk KD, Fröbisch J (2011) A comprehensive taxonomic revision of *Dicynodon* (Therapsida, Anomodontia) and its implications for dicynodont phylogeny, biogeography, and biostratigraphy. Soc Vertebr Paleontol Mem 11: 1-158.

Maisch MW (2001) Observations on Karoo and Gondwana vertebrates. Part 2: A new skull-reconstruction of *Stahleckeria potens* von Huene, 1935 (Dicynodontia, Middle Triassic) and a reconsideration of kannemeyeriiform phylogeny. Neues Jahrb Geol Paläontol Abh 220: 127-152.

Maisch MW (2002) A new basal lystrosaurid dicynodont from the Upper Permian of South Africa. Palaeontol 45: 343-359.

Maisch MW, Gebauer EVI (2005) Reappraisal of *Geikia locusticeps* (Therapsida: Dicynodontia) from the Upper Permian of Tanzania. Palaeontol 48: 309-324.

Modesto SP, Rybczynski N (2000) The amniote faunas of the Russian Permian: implications for Late Permian terrestrial vertebrate biogeography. In: Benton MJ, Shishkin MA, Unwin DM, Kurochkin EN, editors. The age of dinosaurs in Russia and Mongolia. Cambridge: Cambridge University Press. pp. 17-34.

Modesto S, Rubidge B, Welman J (1999) The most basal anomodont therapsid and the primacy of Gondwana in the evolution of the anomodonts. Proc Roy Soc B 266: 331-337.

Modesto S, Rubidge BS, Visser I, Welman J (2003) A new basal dicynodont from the Upper Permian of South Africa. Palaeontol 46: 211-223.

Rubidge BS, Hopson JA (1996) A primitive anomodont therapsid from the base of the Beaufort Group (Upper Permian) of South Africa. Zool J Linn Soc 117: 115-139.

Rybczynski N (2000) Cranial anatomy and phylogenetic position of *Suminia getmanovi*, a basal anomodont (Amniota: Therapsida) from the Late Permian of eastern Europe. Zool J Linn Soc 130: 329-373.

Sidor CA (2001) Simplification as a trend in synapsid cranial evolution. Evolution 55: 1419-1442.

Sidor CA, Hopson JA (1998) Ghost lineages and ‘mammal-ness’: assessing the temporal pattern of character acquisition in the Synapsida. Paleobiol 24: 254-273.

Surkov MV, Benton MJ (2004) The basicranium of dicynodonts (Synapsida) and its use in phylogenetic analysis. Palaeontol 47: 619-638.

Surkov MV, Kalandadze NN, Benton MJ (2005) *Lystrosaurus georgi*, a dicynodont from the Lower Triassic of Russia. J Vertebr Paleontol 25: 402-413.

Vega-Dias C, Maisch MW, Schultz CL (2004) A new phylogenetic analysis of Triassic dicynodonts (Therapsida) and the systematic position of *Jachaleria candelariensis* from the Upper Triassic of Brazil. Neues Jahrb Geol Palaontol Abh 231: 145-166.
